# Coordinate Regulation of Lipid Metabolism by Novel Nuclear Receptor Partnerships

**DOI:** 10.1371/journal.pgen.1002645

**Published:** 2012-04-12

**Authors:** Pranali P. Pathare, Alex Lin, Karin E. Bornfeldt, Stefan Taubert, Marc R. Van Gilst

**Affiliations:** 1 Division of Basic Sciences, Fred Hutchinson Cancer Research Center, Seattle, Washington, United States of America; 2 Molecular and Cellular Biology Program, University of Washington, Seattle, Washington, United States of America; 3 Department of Medicine, Division of Metabolism, Endocrinology, and Nutrition, University of Washington, Seattle, Washington, United States of America; 4 Department of Pathology, Diabetes and Obesity Center of Excellence, University of Washington, Seattle, Washington, United States of America; 5 Department of Medical Genetics, Centre for Molecular Medicine and Therapeutics, University of British Columbia, Vancouver, Canada; 6 Child and Family Research Institute, Vancouver, Canada; University of California San Francisco, United States of America

## Abstract

Mammalian nuclear receptors broadly influence metabolic fitness and serve as popular targets for developing drugs to treat cardiovascular disease, obesity, and diabetes. However, the molecular mechanisms and regulatory pathways that govern lipid metabolism remain poorly understood. We previously found that the *Caenorhabditis elegans* nuclear hormone receptor NHR-49 regulates multiple genes in the fatty acid beta-oxidation and desaturation pathways. Here, we identify additional NHR-49 targets that include sphingolipid processing and lipid remodeling genes. We show that NHR-49 regulates distinct subsets of its target genes by partnering with at least two other distinct nuclear receptors. Gene expression profiles suggest that NHR-49 partners with NHR-66 to regulate sphingolipid and lipid remodeling genes and with NHR-80 to regulate genes involved in fatty acid desaturation. In addition, although we did not detect a direct physical interaction between NHR-49 and NHR-13, we demonstrate that NHR-13 also regulates genes involved in the desaturase pathway. Consistent with this, gene knockouts of these receptors display a host of phenotypes that reflect their gene expression profile. Our data suggest that NHR-80 and NHR-13's modulation of NHR-49 regulated fatty acid desaturase genes contribute to the shortened lifespan phenotype of *nhr-49* deletion mutant animals. In addition, we observed that *nhr-49* animals had significantly altered mitochondrial morphology and function, and that distinct aspects of this phenotype can be ascribed to defects in NHR-66– and NHR-80–mediated activities. Identification of NHR-49's binding partners facilitates a fine-scale dissection of its myriad regulatory roles in *C. elegans*. Our findings also provide further insights into the functions of the mammalian lipid-sensing nuclear receptors HNF4α and PPARα.

## Author Summary

Mammalian nuclear receptors are actively targeted for treatment of a range of cardiovascular diseases and obesity. However, effective drug development still depends on a more exhaustive characterization of how different nuclear receptors mediate their different physiological effects *in vivo*. Taking advantage of the roundworm *Caenorhabditis elegans*, we used a combination of genetic and biochemical approaches to characterize the gene network of the nuclear receptor NHR-49 and to explore the impact of the different target genes on physiology. This work has identified genes and pathways that were not previously known to be regulated by NHR-49. Importantly, we identified NHR-49 co-factors NHR-66 and NHR-80 that regulate specific subsets of NHR-49 target genes and that contribute to distinct phenotypes of *nhr-49* animals. Taken together, our findings in *C. elegans* not only provide novel insights into how nuclear receptor transcriptional networks coordinate to regulate lipid metabolism, but also reveal the breadth of their influence on different aspects of animal physiology.

## Introduction

Modern day lifestyle and diet dramatically increase the threat of chronic diseases including obesity, diabetes and atherosclerosis. These metabolic disorders have been consistently linked to the imbalance between energy consumption and expenditure. Growing evidence suggests that faulty regulation of fat metabolism promotes metabolic diseases [1]. The control of fat metabolism is often mediated by nuclear receptors (NR), which are ligand-regulated transcription factors that play a central role in the cell's ability to sense, transduce and respond to lipophilic signals by modulating the appropriate target genes [2]. Dissecting the role of nuclear receptors in fat metabolism is therefore essential to our understanding of how energy homeostasis is maintained in an organism.

Nuclear receptors typically exhibit highly conserved modular domains including a zinc-finger DNA binding domain (DBD) and a ligand-binding domain (LBD) [2]. Ligand binding affects nuclear receptor activity by inducing structural changes within the LBD, which then alters the receptor's affinity to different co-factor proteins such as co-regulators and binding partner(s). Co-regulators include both co-activators and co-repressors, and are critical in mediating transcriptional responses. Alteration in co-regulator–NR binding can thus lead to a change in the transcriptional response. For instance, co-repressors can be replaced with co-activators to promote transcription of target genes, or NRs can form distinct homo- or heterodimers to activate or repress a specific set of target genes [3]. Thus, binding of distinct co-factors determines how nuclear receptors influence different gene networks.

The Hepatocyte Nuclear Factor 4- alpha (HNF4α) is an example of a lipid sensing nuclear receptor in mammals. HNF4α orchestrates the regulation of a diverse range of target genes and is especially important in the control of genes involved in glucose and fatty acid homeostasis [4], and is mainly expressed in the liver, pancreas, kidney and small intestine [5]. Consistent with its role in metabolism, mutations in HNF4α are associated with both maturity onset diabetes of the young (MODY) and type 2 diabetes [6]–[9]. Although it is not entirely clear how HNF4α protects against diabetes, it has been reported that mutations in HNF4α can lead to early death of pancreatic beta-cells resulting in pancreatic dysfunction and a subsequent decrease in insulin production [10], [11]. In addition, knock-out of HNF4α in the adult mouse liver leads to increased lipid accumulation in hepatocytes and to the misregulation of genes involved in glucose and lipid metabolism [12], [13].

HNF4 receptors are evolutionarily conserved. Whereas mammals have two paralogs of this receptor, HNF4α and HNF4γ, the HNF4 family has expanded enormously in the nematode *C. elegans* to include 269 HNF4-like members [14], [15]. One of these 269 HNF4-like members is NHR-49, which is remarkably similar to the mammalian Peroxisome Proliferator-Activated Receptors (PPARs) in its overall biological effects on metabolism, fat storage, and life span [16]. The PPARs are global modulators of fat metabolism, controlling fat storage, expenditure, distribution and transport [17]. Perhaps most striking is the finding that NHR-49 and the PPARs, particularly PPARα and PPARδ, positively influence similar genes in multiple metabolic processes, including fatty acid β-oxidation, fatty acid desaturation, and fatty acid binding/transport [17], [18]. Moreover, knockout of PPARα or PPARδ can lead to high-fat phenotypes [18], [19] that are similar to those observed in *nhr-49* animals, demonstrating the common physiological effects of these receptors on fat storage.

In this study, we set out to elucidate the genome-wide regulatory network of NHR-49 and to characterize its target genes to better understand the impact of NHR-49 mediated transcriptional regulation on worm physiology. We identified NHR-66 and NHR-80 as physical NHR-49 co-factors and were able to delineate their specific contribution to distinct phenotypes of *nhr-49* mutants, namely selective effects of individual co-factors on life span and on mitochondrial function. We also uncovered novel roles for NHR-49 in the regulation of lipid metabolism including sphingolipid breakdown and lipid remodeling. Taken together, our findings support a model whereby NHR-49 heterodimerizes with other nuclear receptors to mediate the activation or repression of genes involved in distinct aspects of lipid metabolism.

## Results

### NHR-49 activates and represses genes in multiple lipid metabolism pathways

We previously found that NHR-49 promotes two distinct aspects of lipid metabolism, fatty acid desaturation and fatty acid β-oxidation [16]. However, the complete list of NHR-49's regulatory targets was still not known. Thus, we used whole genome *C. elegans* oligonucleotide microarrays to define the transcriptional profiles in an *nhr-49(nr2041)* deletion strain (compared to N2 wild-type worms). Table 1 lists genes that exhibit statistically significant changes in expression with (|log_2_(ratio)|≥0.848 and p-value≤0.001), in *nhr-49* animals ([20] and Materials and Methods). The genes with negative values of logFC (fold change) are down regulated in the *nhr-49* mutant (i.e. activated by NHR-49), whereas the genes with positive values of logFC are up regulated in the *nhr-49* mutant (repressed by NHR-49). The finding that *acs-2*, which participates in mitochondrial β-oxidation and was previously demonstrated to be an NHR-49 target gene [16] had reduced expression in *nhr-49* mutants validated the experimental approach. However, some of the previously reported genes like *fat-5*, *fat-6*, *fat-7*, *cpt-2* and *ech-1* [16], were not found to be significant in our microarray analysis. This is likely due to the cut-off that we employed. We also uncovered new targets that are repressed by NHR-49 that include genes involved in sphingolipid breakdown, lipid remodeling and xenobiotic detoxification.

**Table 1:** List of differentially regulated genes in *nhr-49* compared to wild-type animals.

ID	Name	logFC	AveExpr	P.Value	Gene WB ID	Gene Public Name
cea2.p.158395	F45D11.1	2.36	8.5	3.84E-05	WBGene00018448	F45D11.1
cea2.i.07091	Y71F9B.6	1.18	6.75	0.000976	WBGene00022128	Y71F9B.6
cea2.i.11595	F27E5.1	2.55	8.52	2.90E-05	WBGene00009192	F27E5.1
cea2.p.110908	C45B11.3	−2.83	7.56	0.000249	WBGene00000981	dhs-18
cea2.i.31736	ZK617.2	2.33	11.1	8.42E-05	WBGene00014009	lips-6
cea2.3.07963	E02H9.5	2.41	6.7	1.94E-05	WBGene00017103	E02H9.5
cea2.i.08369	B0454.8	1.75	7.7	0.000706	WBGene00015199	B0454.8
cea2.i.31956	B0222.4	4.12	6.42	6.03E-05	WBGene00006418	tag-38
cea2.d.10890	C05E4.9b	−1.57	11.4	0.000505	NA	NA
cea2.i.48948	ZK218.5	2.35	9.57	5.54E-05	WBGene00013939	ZK218.5
cea2.p.110949	C47A10.1	2.07	8.23	5.08E-05	WBGene00004003	pgp-9
cea2.i.07568	ZK265.1	−1.33	8.32	0.00053	WBGene00013955	kri-1
cea2.i.32048	B0348.2	4.36	8.64	2.67E-06	WBGene00015152	B0348.2
cea2.p.48441	W02B12.1	1.61	9.77	0.000192	WBGene00012201	W02B12.1
cea2.c.41613	C18A11.1	1.78	8.19	0.000508	WBGene00015947	C18A11.1
cea2.d.33151	K09H11.7	−1.52	11.2	0.000274	WBGene00019604	K09H11.7
cea2.c.35812	F28F8.2	−4.44	8.05	9.88E-07	WBGene00009221	acs-2
cea2.d.21244	F18E3.7a	−1.48	7.09	0.000358	NA	NA
cea2.p.120260	K01D12.11	1.52	10.5	0.000667	WBGene00010470	cdr-4
cea2.i.30276	Y46C8AL.3	1.56	6.73	0.00046	WBGene00021581	clec-70
cea2.p.47717	T24E12.5	2.53	6.62	0.000225	WBGene00020774	T24E12.5
cea2.p.87235	C32H11.12	−1.73	7.33	0.000163	WBGene00007875	dod-24
cea2.c.15603	Y57A10C.6	−1.3	10.2	0.000595	WBGene00013284	Y57A10C.6
cea2.p.114229	F21F8.4	1.54	9.94	0.000793	WBGene00017678	F21F8.4
cea2.d.21251	F18E3.7a	−1.89	8.97	8.26E-05	NA	NA
cea2.i.33937	C29F3.5	2.74	7.36	0.000224	WBGene00007806	clec-230
cea2.p.47251	T19D12.4	1.92	7.68	0.000372	WBGene00020579	T19D12.4
cea2.d.21252	F18E3.7a	−2	9.62	5.71E-05	NA	NA
cea2.i.46562	Y37H2A.11	2.69	9.5	0.000213	WBGene00012571	Y37H2A.11
cea2.p.21595	Y47H10A.5	1.59	9.24	0.000476	WBGene00012961	Y47H10A.5
cea2.p.40219	F49E12.9	1.61	8.34	0.000365	WBGene00009902	F49E12.9
cea2.i.44641	T16G1.6	1.91	7.4	0.000298	WBGene00011800	T16G1.6
cea2.p.39387	F43C11.7	1.35	7.14	0.000667	WBGene00018384	F43C11.7
cea2.d.24252	F37B1.3	1.86	7.32	0.000357	WBGene00001762	gst-14
cea2.c.32957	ZK550.6	−1.3	9.65	0.000807	WBGene00014000	ZK550.6
cea2.i.15206	T10B9.1	2.1	7.82	5.66E-05	WBGene00011671	cyp-13A4
cea2.i.46998	Y40B10A.2	1.4	8.39	0.000517	WBGene00021487	Y40B10A.2
cea2.i.38305	F35E8.8	1.92	7.36	0.000136	WBGene00001786	gst-38
cea2.i.35082	C53A3.2	−1.58	8.31	0.000314	WBGene00016892	C53A3.2
cea2.i.24912	C54E4.5	1.47	8.8	0.000379	WBGene00016920	C54E4.5
cea2.i.34217	C36C5.14	2.15	7.07	6.50E-05	WBGene00016483	C36C5.14
cea2.p.110129	C29F3.2	2.28	7.58	6.65E-05	WBGene00006954	wrt-8
cea2.c.38150	T05B4.3	−1.33	11.1	0.000641	WBGene00020237	phat-4
cea2.p.117903	F53E10.4	1.54	7.15	0.000519	WBGene00018760	F53E10.4
cea2.d.42799	Y19D10A.9	−2.02	7.77	0.000151	WBGene00021224	clec-209
cea2.i.06720	Y65B4BR.1	2.78	10.1	0.000136	WBGene00022040	Y65B4BR.1
cea2.c.37571	K12G11.3	−2.63	9.5	4.30E-05	WBGene00010790	sodh-1

**Figure 1:**
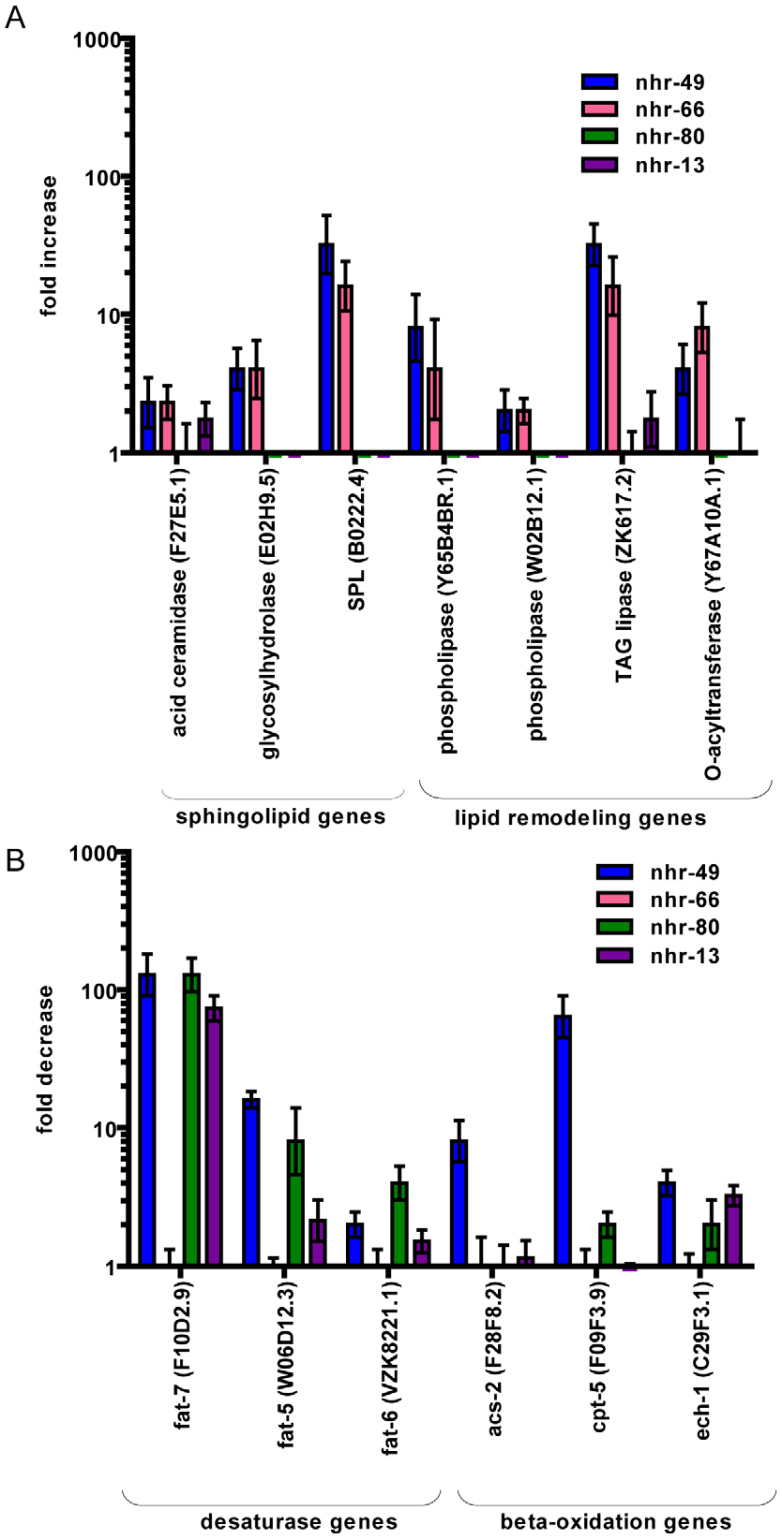
NHR-49 collaborates with NHR-66 to regulate sphingolipid and lipid remodeling genes and with NHR-80 and NHR-13 to regulate fatty acid desaturase genes. (A) qRT-PCR measurement of genes involved in sphingolipid metabolism including fatty acid ceramidase, sphingosine phosphate lyase, glycosyl hydrolase and genes involved in lipid remodeling namely phospholipases, TAG lipase and O-acyltransferease expression in *nhr-49(nr2041)* (blue bars), *nhr-66(ok940)* (pink bars), *nhr-80(tm1011)* (green bars) and *nhr-13(gk796)* (purple bars) relative to wild-type animals. Values represent overall fold change of *nhr-49(nr2041)*, *nhr-66(ok940)*, *nhr-80(tm1011)* and *nhr-13(gk796)* animals with respect to wild-type worms. Expression was measured in L4 stage of development. Error bars represent SEM from 3 independent experiments. (B) qRT-PCR measurement of genes involved in fatty acid desaturases including *fat-5*, *fat-6* and *fat-7* and genes involved in fatty acid beta-oxidation including *acs-2*, *cpt-5* and *ech-1* in *nhr-49(nr2041)* (blue bars), *nhr-66(ok940)* (pink bars), *nhr-80(tm1011)* (green bars) and *nhr-13(gk796)* (purple bars) relative to wild-type animals. Values represent overall fold change in *nhr-49(nr2041)*, *nhr-66(ok940)*, *nhr-80(tm1011)* and *nhr-13(gk796)* animals relative to wild-type worms. Expression was measured at L4 stage of development. Error bars represent SEM from 3 independent experiments.

To validate these candidate NHR-49 targets, we employed quantitative RT-PCR to analyze their mRNA levels (Figure 1). In *nhr-49* mutants, the expression of the sphingolipid processing genes acid ceramidase, glycosyl hydrolase, sphingosine-phosphate lyase (SPL), as well as lipid remodeling genes like phospholipases, TAG lipase and O-acyltransferase, was up regulated, as expected (Figure 1A and Table S1). Consistent with previously published data, *nhr-49* mutants also showed a decrease in the expression of the fatty acid desaturase genes *fat-7*, *fat-5* and *fat-6*, and the fatty acid beta-oxidation genes *acs-2*, *cpt-5* and *ech-1* (Figure 1B). Together, the gene expression data confirm that NHR-49 activates fatty acid β-oxidation and desaturase genes, and for the first time identifies target genes repressed by NHR-49 that include those involved in sphingolipid processing and lipid remodeling.

**Figure 2:**
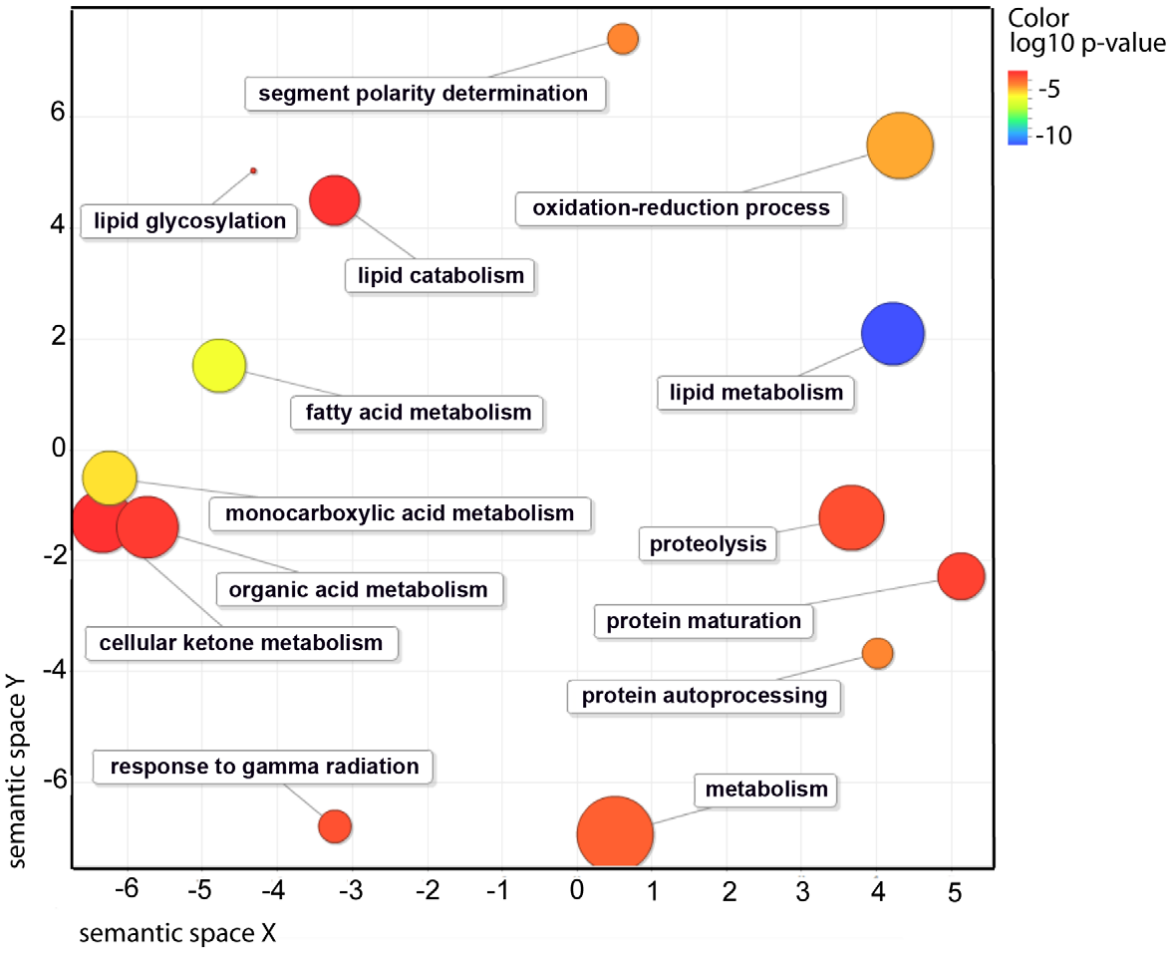
Functional classification summary for the *nhr-49* mutant are represented as a scatter plot using the GO visualization tool REViGO. X- and Y-axes represent a two-dimensional annotation space derived from a multi-dimensional scaling procedure used on a matrix of GO terms' semantic similarities (see reference 51 for details). By employing this visualization method, similar functional categories will cluster together. Bubble color represents the p-value obtained from GO term enrichment analysis (GOrilla, see Materials and Methods) and bubble size relates to the frequency of GO terms in the Gene Ontology Annotation Database.

To identify pathways and molecular functions common to the genes observed by microarray analysis, we employed the gene ontology (GO) enrichment analysis using GOrilla [21]. As expected due to NHR-49's known role in lipid biology, there was a significant overrepresentation of GO-terms for functions related to fat metabolism (Figure 2 and Table 2). We also found that pathways regulating protein processing, maturation and proteolysis were overrepresented.

**Table 2:** Occurrence of gene families in microarray results for NHR-49, based on GO-terms.

GO Term	Description	P-value
GO:0006629	lipid metabolic process	1.55E-11
GO:0006631	fatty acid metabolic process	1.45E-06
GO:0032787	monocarboxylic acid metabolic process	4.46E-06
GO:0044255	cellular lipid metabolic process	1.34E-05
GO:0055114	oxidation-reduction process	1.72E-05
GO:0016540	protein autoprocessing	3.87E-05
GO:0007367	segment polarity determination	3.87E-05
GO:0007365	periodic partitioning	3.87E-05
GO:0030258	lipid modification	4.28E-05
GO:0008152	metabolic process	1.52E-04
GO:0010332	response to gamma radiation	2.66E-04
GO:0006508	proteolysis	2.87E-04
GO:0016485	protein processing	3.52E-04
GO:0034440	lipid oxidation	3.84E-04
GO:0006635	fatty acid beta-oxidation	3.84E-04
GO:0019395	fatty acid oxidation	3.84E-04
GO:0072329	monocarboxylic acid catabolic process	3.84E-04
GO:0009062	fatty acid catabolic process	3.84E-04
GO:0051604	protein maturation	4.60E-04
GO:0030259	lipid glycosylation	4.91E-04
GO:0043436	oxoacid metabolic process	6.05E-04
GO:0006082	organic acid metabolic process	6.05E-04
GO:0019752	carboxylic acid metabolic process	6.05E-04
GO:0016042	lipid catabolic process	8.12E-04
GO:0044242	cellular lipid catabolic process	8.83E-04
GO:0042180	cellular ketone metabolic process	8.95E-04

### Identification of candidate NHR-49 co-factors

Because NRs form homo and/or heterodimers and associate with co-regulators, we hypothesized that NHR-49 might differentially regulate its distinct target genes by interacting with specific transcriptional co-factors [22]. To identify such factors, we performed a yeast two-hybrid screen using the NHR-49-LBD as bait. In addition to the Mediator subunit MDT-15 [23], we identified six NHRs as candidate NHR-49-LBD partners (Table S2a). Notably, all NHR prey clones identified included their respective LBDs, suggesting that dimerization occurs via the LBD, as has been described for other NRs. We estimated the relative binding strength of these preys using a LacZ reporter, and found that five of the six NHRs interacted strongly and specifically with the NHR-49 LBD (Table S2b). In parallel, a large-scale yeast-two-hybrid screen identified a set of 13 additional candidates to interact with full-length NHR-49 (Table S2a) [24]. Because they were identified in both yeast-two-hybrid screens, we deemed NHR-13, MDT-15, NHR-22, NHR-66, and NHR-105 as most likely to represent NHR-49 cofactors.

### Specific co-factors regulate distinct NHR-49 pathways

To determine whether any of these cofactors regulate the newly identified NHR-49 pathways of sphingolipid processing, lipid remodeling or the β-oxidation and fatty acid desaturation pathways, we quantified the mRNA levels of the NHR-49 activated and repressed genes in these pathways (see primer pairs, Tables S3 and S4). We chose to analyze the *nhr-13*(*gk796*) and *nhr-66*(*ok940*) mutants, and also included *nhr-80(tm1011)* because it regulates the fatty acid desaturase genes [25]. Our qRT-PCR analyses revealed a striking separation of gene regulation by the different candidate co-factors. The deletion of *nhr-66(ok940)* resulted in the up regulation of most of the genes that are repressed by NHR-49, including the sphingolipid and lipid remodeling genes (Figure 1A and Table S1). The levels of derepression of these genes observed in *nhr-66* mutants were comparable to that seen in *nhr-49* animals, suggesting that the two NRs act together. In contrast, *nhr-66* mutants did not show any change in the expression of *nhr-49* activated genes, like those involved in β-oxidation and desaturation (Figure 1B and Table S1). These results strongly suggest that NHR-49 acts with NHR-66 to specifically repress the transcription of sphingolipid and lipid remodeling genes.

In contrast, *nhr-80* and *nhr-13* deletion mutants do not affect the sphingolipid, lipid remodeling, or β-oxidation genes. Instead, these mutants exhibited a decreased expression of the fatty acid desaturase genes *fat-7*, *fat-5* and *fat-6* (Figure 1B), confirming previous analyses of *nhr80* mutants by the Watts laboratory [25]. The *fat-5*, *fat-6* and *fat-7* genes are members of the Δ9 fatty acid desaturases and are key enzymes in fatty acid metabolism [26]. Their function is to introduce a double bond in saturated fatty acid chains to generate monounsaturated fatty acids (MUFAs) that are important in membrane fluidity and energy storage [27]. These results suggest that NHR-49 associates with NHR-80 and NHR-13 to regulate the ratio of saturated and unsaturated fat in lipid membranes. However, the fold changes observed in fatty acid desaturation regulation by NHR-80 and NHR-13 did not parallel those observed in *nhr-49* mutants, suggesting the involvement of other regulatory factors. Consistent with this, the *C. elegans* ortholog of the sterol-regulatory-element-binding protein (SREBP) transcription factor SBP-1 and the Mediator subunit MDT-15 are also implicated in the regulation of fatty acid desaturases [23], [28]–[30].

### NHR-66 and NHR-80 regulate additional targets independently of NHR-49

**Figure 3:**
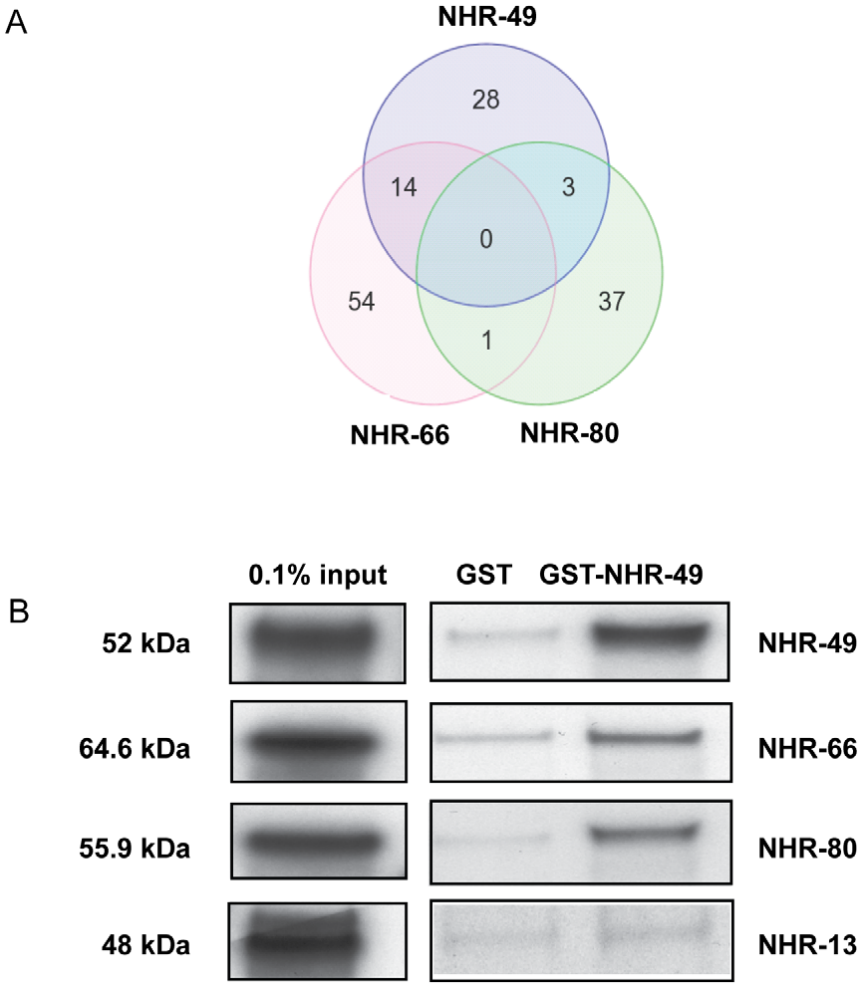
NHR-49 shares target genes and physically interacts with NHR-66 and NHR-80. (A) Venn diagram showing the comparison of genes regulated by NHR-49, NHR-66 and NHR-80. Gene identities are presented in Table S9. (B) NHR-49 physically interacts with NHR-49, NHR-66 and NHR-80 *in vitro*. Pull-down assays showing in vitro translated ^35^S-methionine labeled NHR-49, NHR-66, NHR-80 and NHR-13 binding purified GST-NHR-49 fusion protein versus purified GST-alone control. The protein mixtures of ^35^S-methionine-labeled NHRs were incubated with glutathione-sepharose 4B beads and analyzed by SDS-PAGE (4–12%) followed by autoradiography. The input represents 0.1% of the labeled proteins used for the pull down assays.

Our data strongly suggest that NHR-66 regulates genes in the sphingolipid and lipid remodeling pathways whereas NHR-80 regulates the fatty acid desaturase genes. To determine whether these NRs regulate other genes in addition to these NHR-49 targets, we performed a genome-wide microarray analysis on *nhr-66* and *nhr-80* mutants. The list of differentially regulated *nhr-66* and *nhr-80* genes is presented in tables S5 and S7, respectively, and the corresponding GO term analyses of NHR-66 and NHR-80 is presented in Tables S6 and S8, respectively. As expected, the microarray data confirmed that NHR-66 regulates several genes involved in the sphingolipid and lipid remodeling pathways, and that NHR-80 regulates genes involved in fatty acid desaturation. In addition, Figure 3A and Table S9 show the genes commonly regulated by NHR-49 & NHR-66 and NHR-49 & NHR-80, using our microarray analysis. We also found evidence that these nuclear receptors regulate additional target genes. For example, NHR-66 is involved in GDP-mannose metabolic processes and regulates *gmd-2*, a GDP-mannose dehydratase. The NHR-80 GO analysis indicates that it regulates genes involved in several different processes including embryonic development and cell death. In conclusion, our analyses suggest that NHR-66 and -80 are not only transcriptional partners required for NHR-49, but can independently act with other binding partners to regulate unique pathways (Figure 3A).

### NHR-49 physically interacts with NHR-66 and NHR-80

Our yeast-two-hybrid and gene expression data suggested that NHR-49 might directly interact with NHR-66, NHR-80 and NHR-13. To test this, we performed *in vitro* GST-NHR-49 pull-down assays with full-length NHR-66, NHR-80 and NHR-13 proteins. NHR-49 was a positive control since it exhibits a two-hybrid interaction with itself [24]. Purified glutathione-S-transferase (GST)-tagged NHR-49 was incubated with *in vitro* translated ^35^S-methionine-labeled NHR-49, NHR-66, NHR-80 and NHR-13. As expected, GST-NHR-49 successfully formed a homodimer with radiolabeled NHR-49 (Figure 3B). In addition, it was also able to interact directly with NHR-66 and NHR-80. NHR-13 did not bind to NHR-49 *in vitro* above background levels, suggesting that additional factors may contribute to an interaction with NHR-49 *in vivo*. Alternately, NHR-13 may regulate fatty acid desaturation via another unknown mechanism.

### NHR-49's regulation of fatty acid desaturases contributes to lifespan

The physical interaction and functional studies support a model whereby the control of distinct NHR-49 regulatory modules is based on NHR-49's association with distinct partner proteins. In this case, mutation of individual NHR-49 partners should delineate the contributions of each co-factor to the phenotypes observed in *nhr-49* mutants. For example, knocking out NHR-66 could reveal NHR-49-dependent sphingolipid and lipid remodeling gene mediated phenotypes, whereas knocking out NHR-80 and NHR-13 could reveal NHR-49 dependent fatty acid desaturase regulated phenotypes. We note that although we did not obtain clear evidence that NHR-13 is a direct physical partner of NHR-49, we continued to characterize it because its gene expression profile suggests it plays a role in fatty acid desaturation.

**Figure 4:**
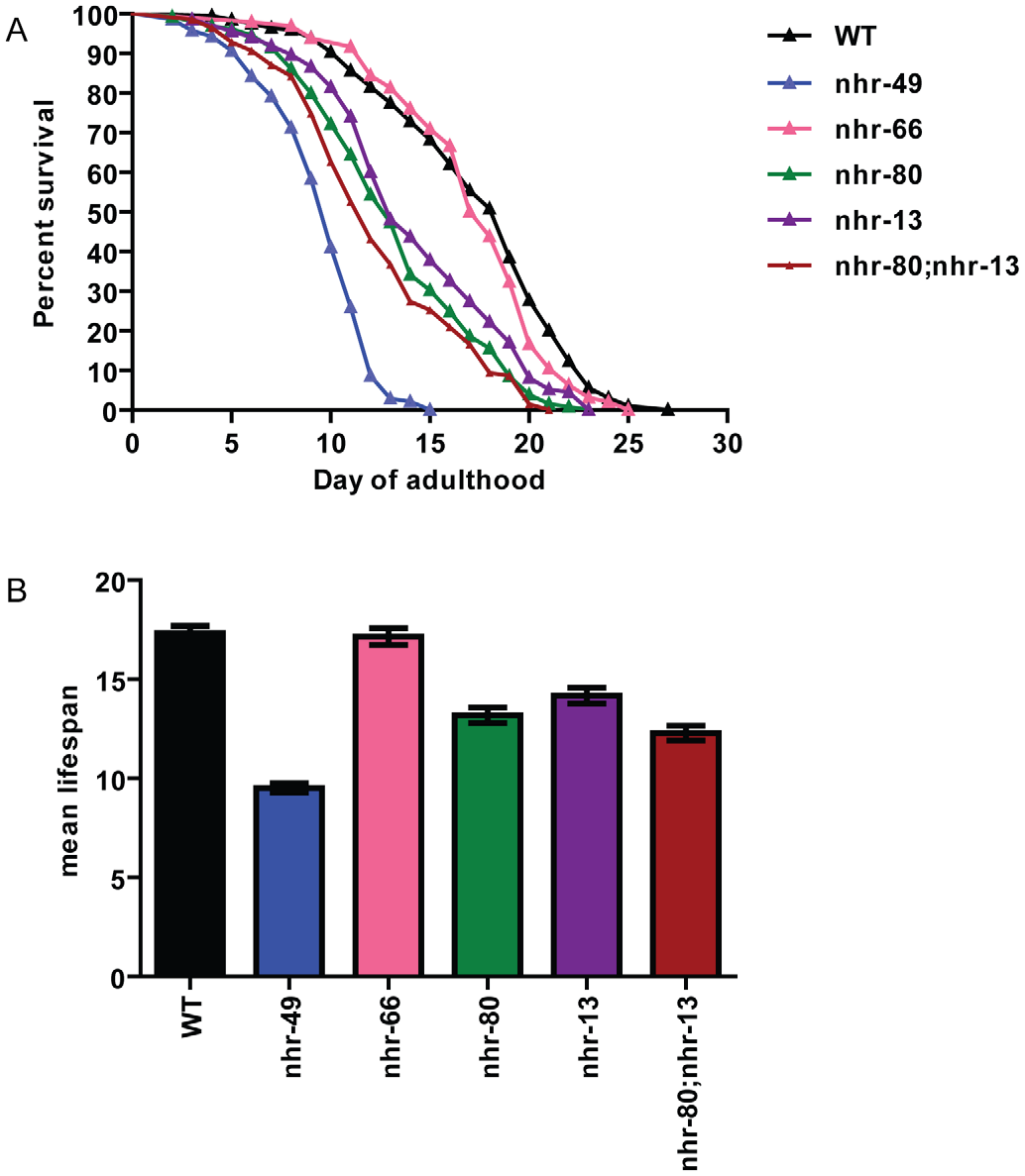
*nhr-80; nhr-13* double mutants have a reduced lifespan comparable to that of *nhr-49* animals. (A) The percentage of animals remaining alive is plotted against animal age. Adult lifespan survival curves of WT (black), *nhr-49(nr2041)* (blue), *nhr-66(ok940)* (pink), *nhr-80(tm1011)* (green), *nhr-13(gk796)* (purple) and double mutant *nhr-80; nhr-13* (brown) animals. (B) The bar graph represents the combined mean (+/−SEM) adult lifespan (days) from at least four independent experiments.

Among the many phenotypes exhibited by *nhr-49* mutants, we chose to focus on its reduced lifespan. To determine what pathway of NHR-49 is important in regulating its lifespan, we analyzed the individual co-factor mutants for effects on lifespan. The *nhr-66* mutant animals had a lifespan of 17.16+/−0.41 days compared to wild-type lifespan of 17.35+/−0.34 days, suggesting that sphingolipid and lipid remodeling genes do not play a role in the reduced lifespan phenotype of *nhr-49* mutants (Figure 4). In contrast, at 20°C, *nhr-80* and *nhr-13* mutants had significantly shorter lifespans of 13.19+/−0.38 days and 14.17+/−0.4 days, respectively (Figures 4A and 4B and Table S10). Moreover, an *nhr-80; nhr-13* double mutant had a lifespan of 12.29+/−0.37 days, which approaches the *nhr-49* mutant lifespan of 9.52+/−0.23 days. Together, these results suggest that the reduced expression of fatty acid desaturases observed in the *nhr-80* and *nhr-13* mutants might contribute to the shortened lifespan of *nhr-49* animals.

**Figure 5:**
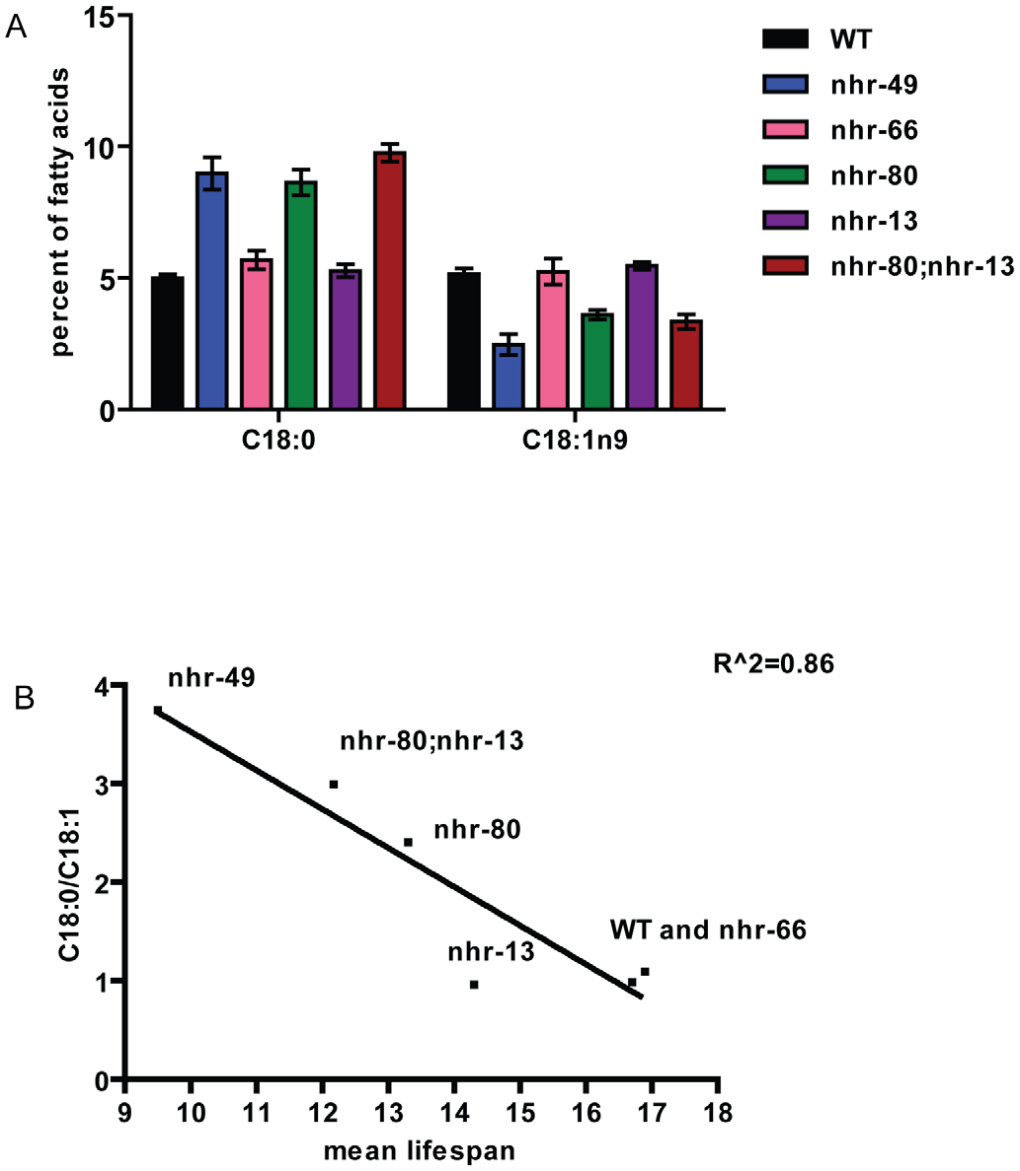
Higher levels of saturated fat in *nhr-80;nhr-13* and *nhr-49* animals correlate with shortened lifespans. (A) Relative abundance of individual fatty acid species expressed as percentage of total measured fatty acid. Fatty acids were isolated and quantified by GC/MS from WT (black bars), *nhr-49* (blue bars), *nhr-66* (pink bars), *nhr-80* (green bars), *nhr-13* (purple bars) and *nhr-80; nhr-13* (brown) animals. Error bars represent SEM. (B) Correlation between the ratio of C18:0 to C18:1n9 and mean lifespan.

These observations are in accordance with previously published work demonstrating that shortened lifespan is correlated with lowered expression of the *fat-7* SCD (Stearoyl CoA Desaturase) [16]. In addition, both *nhr-49* mutants and worms where FAT-7 was knocked down using RNA-interference, exhibit a concomitant increase in the levels of saturated fat. More specifically, the reduction in desaturase levels strongly alter the ratio between stearic acid and oleic acid (C18:0/C18:1n9) to approximately 3.74+/−0.33 in *nhr-49* animals (0.98+/−0.06 in wild-type animals). We thus quantified the abundance of individual fatty acids species in *nhr-13*, *nhr-80*, and *nhr-80; nhr-13* mutants using Gas Chromatography-Mass Spectrometry (GC-MS). The C18:0/C18:1n9 ratio in *nhr-80; nhr-13* double mutants (2.99+/−0.22) was similar to the ratio observed in *nhr-49* mutants, and higher than the ratio in wild-type worms (0.98+/−0.06) (Figure 5A and Table S11). Strikingly, there is a strong inverse correlation (r^2^ = 0.86) between the level of saturated fat and the duration of mean worm lifespan (Figure 5B), supporting the notion that excess saturated fat plays a role in the early death of the *nhr* mutants. These results also suggest that NHR-49's interaction with NHR-80 and possibly NHR-13 contributes to the shortened lifespan phenotype that is observed in *nhr-49* mutants.

### NHR-49 is important for normal mitochondrial morphology

**Figure 6:**
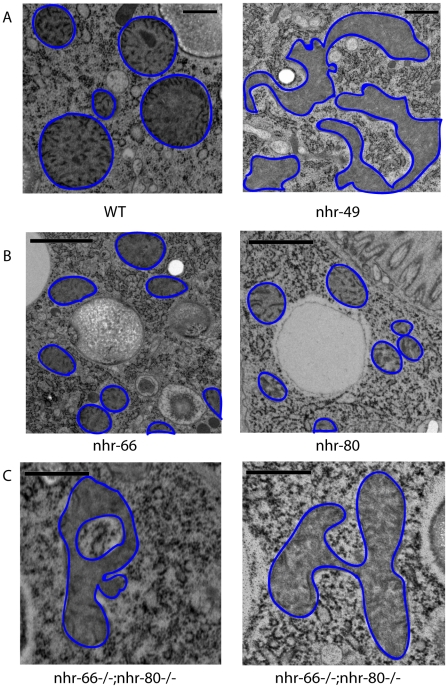
*nhr-49* mutants show abnormal mitochondrial morphology. (A) High pressure freezing transmission electron microscopy (HP-TEM) images of wild-type animals and *nhr-49* mutants at the same magnification. Note that mitochondria are outlined in blue. Bar, 0.5 µm. (B) HP-TEM images of *nhr-66* and *nhr-80* mutants at the same magnification. Note that the mitochondria are outlined in blue. Bar, 1.0 µm. (C) Two HP-TEM images of *nhr-66; nhr-80* animals at the same magnification. Note that the mitochondria are outlined in blue. Bar, 1.0 µm.

**Figure 7:**
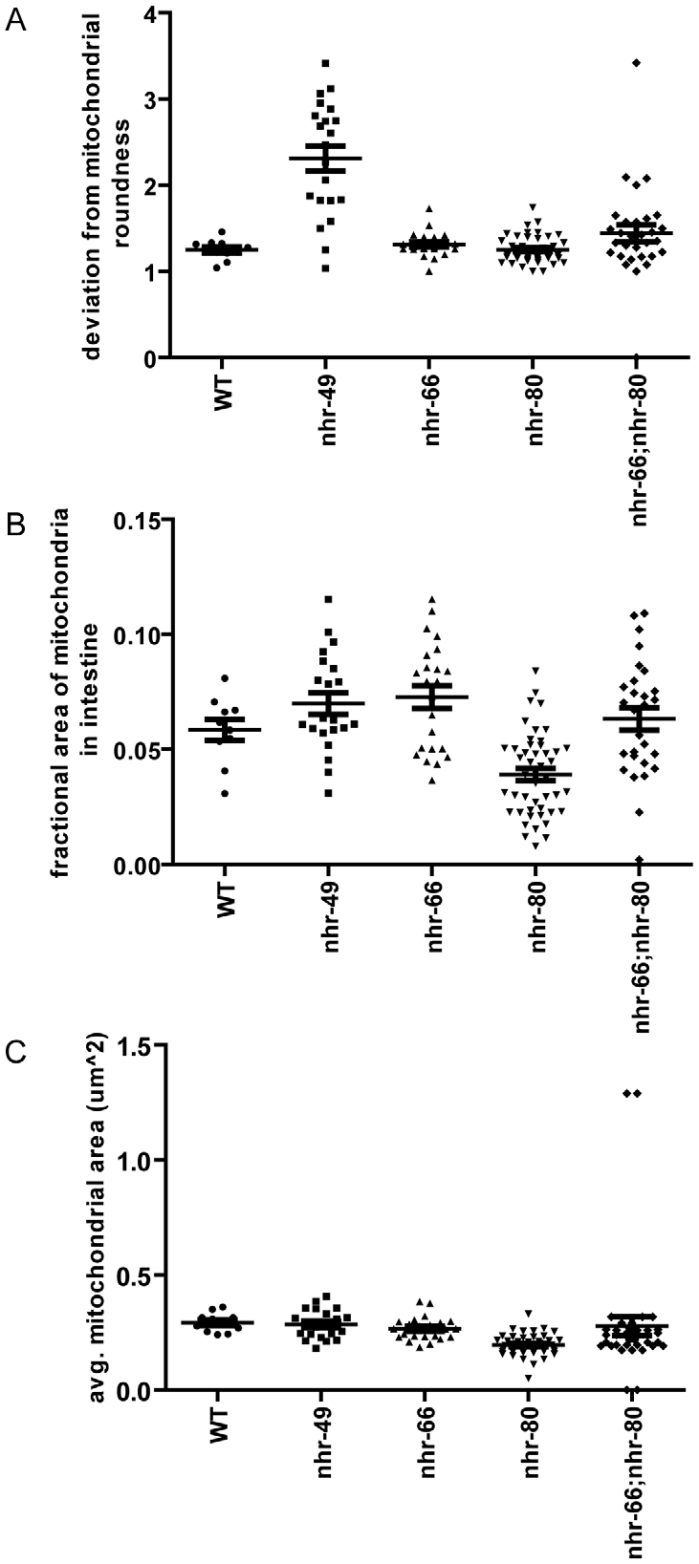
*nhr-49*, *nhr-66*, and *nhr-80* animals have abnormal mitochondrial phenotypes. (A) Quantification of mitochondrial size in WT, *nhr-49*, *nhr-80*, *nhr-66* and *nhr-66; nhr-80* animals. Each point represents the average size of all the intestinal mitochondria examined per section. P-values were obtained using the unpaired t-test with Welch's correction for unequal variance. This analysis showed that the difference between *nhr-80* animals and wild-type is statistically significant with a p-value<0.0001. (B) Quantification of mitochondrial fractional area in WT, *nhr-49*, *nhr-80*, *nhr-66* and *nhr-66; nhr-80* animals. Each point represents the average fractional area (µm^2^) of the examined mitochondria per section. P-values were obtained using the unpaired t-test with Welch's correction for unequal variance. This analysis showed that the difference between *nhr-66* and *nhr-80* animals compared to wild-type was statistically significant with p-values of 0.0440 and 0.0025 respectively. (C) Quantification of mitochondrial shape in WT, *nhr-49*, *nhr-80*, *nhr-66* and *nhr-66; nhr-80* animals. Each point represents the average number of mitochondrial turns in the section examined. P-values were obtained using the unpaired t-test with Welch's correction for unequal variance. This analysis showed that the difference between *nhr-49*animals compared to wild-type was statistically significant with a p-value<0.0001.

To understand why the imbalance of lipid saturation observed in *nhr-49* mutants could be detrimental to the animal, we hypothesized that the excess pool of saturated fat might get incorporated into membranes, thus affecting their function. Because NHR-49 functionally resembles the PPAR family of mammalian nuclear receptors that promote mitochondrial biogenesis [31], [32], we employed high pressure-transmission electron microscopy (HP-TEM) to visualize mitochondria in *nhr-49* animals (Figure 6). This ultrastructural analysis revealed multiple morphological defects in the intestinal mitochondria of one-day old adult *nhr-49* mutants (compared to wild-type worms), although mitochondria in *nhr-49* mutants were comparable in size to wild-type mitochondria (Figure 7A). Strikingly, when we measured the average fractional area occupied by mitochondria per total intestinal area in *nhr-49* mutants, we found that there was a considerable variation in the distribution, although the average was not statistically different from wild-type animals (Figure 7B). Moreover, about 25% of the intestinal mitochondria of the *nhr-49* mutants were uncharacteristically irregular in shape and appeared to have more turns compared to wild-type animals (Figure 6A). The average number of turns exhibited by the intestinal mitochondria in *nhr-49* animals was greater than the more rounded wild-type mitochondria (Figure 7C). Taken together, these results suggested that mitochondria in *nhr-49* animals exhibit an altered shape when compared to those in wild-type animals of the same age.

### Partner co-factors contribute to NHR-49's regulation of mitochondrial morphology

We next asked if the NHR-49 partners NHR-66 and NHR-80 contribute to the observed NHR-49 mitochondrial abnormalities. In contrast to *nhr-49* mutants, the mitochondria in one-day old adult *nhr-66* and *nhr-80* mutants were comparable in shape to those observed in wild-type worms (Figure 6B and 7A). However, the average fractional area occupied by the mitochondria in the intestine was significantly higher in *nhr-66* animals and significantly lower in *nhr-80* animals (compared to wild-type worms; Figure 7B). Lastly, even though the average size of the intestinal mitochondria in the *nhr-66* animals was similar to the size of mitochondria in wild-type animals, the *nhr-80* animals showed significantly smaller mitochondria (Figure 7C).

Because these data suggested that NHR-66 and NHR-80 contribute to mitochondrial morphology, we next examined intestinal mitochondria in one-day old adult *nhr-66; nhr-80* double mutants. The fractional area of mitochondria in these animals was not statistically different from wild-type when averaged, but the distribution was much broader, similar to *nhr-49* animals (Figure 7B). In addition, 11.4% of the intestinal mitochondria in the double mutants were highly irregular in shape, as was observed in 25% of the mitochondria in *nhr-49* mutants (Figure 6C and Figure 7C). Although the mitochondrial phenotypes in *nhr-66; nhr-80* mutants are not as strong as those in the *nhr-49* animals, these data do suggest that NHR-66 and NHR-80 contribute to normal mitochondria morphology. Together, these data suggest that NHR-49 maintains mitochondrial morphology via multiple pathways, including NHR-66 and NHR-80 dependent regulation as well as additional, unknown mechanisms.

### Impaired mitochondrial morphology affects physiological function

**Figure 8:**
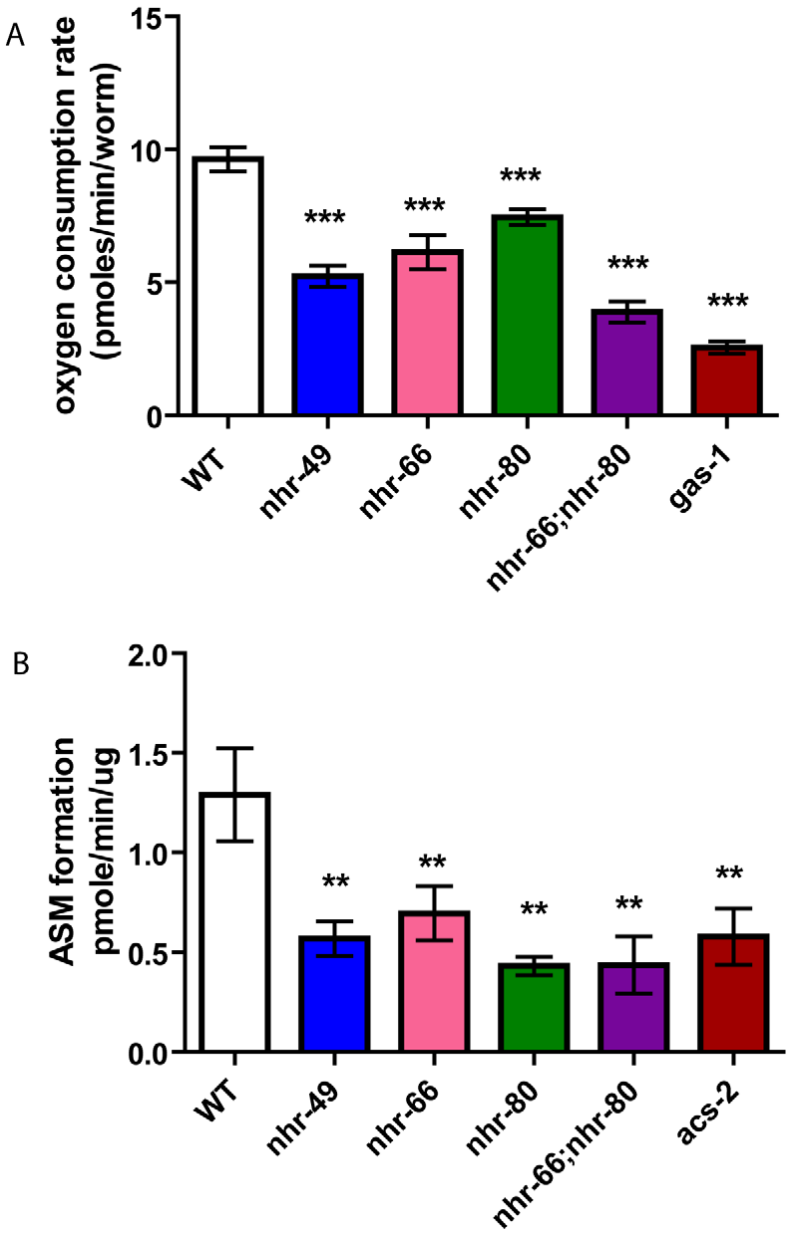
*nhr-49* animals have reduced basal oxygen consumption rates and reduced beta-oxidation. (A) Basal oxygen consumption rates of WT, *nhr-49*, *nhr-80*, *nhr-66*, *nhr-66; nhr-80* and *gas-1* animals. P-values were obtained using the unpaired t-test. *** p<0.001. (B) Evaluating β-oxidation by measuring amounts of radiolabeled acid-soluble metabolites in WT, *nhr-49*, *nhr-80*, *nhr-66*, *nhr-66; nhr-80* and *acs-2* animals. The data represents the average results from two independent experiments. The results are expressed as means +/− SEM (n = 6) normalized to the specific activity of the radioactive palmitate. One-way ANOVA was used to compare with the Neuman-Keuls multiple comparison tests. **<p<0.01, *** p<0.001.

Because mitochondrial morphology was altered in the *nhr* mutants, we assayed mitochondrial function using two metabolic assays. First, we monitored the basal oxygen consumption rates in live animals (normalized to worm count). Consistent with the mitochondrial abnormalities observed by electron microscopy, *nhr-49* animals consumed oxygen at significantly reduced rates of 5.22 pmoles/min/worm compared to 9.625 pmoles/min/worm in wild-type animals (Figure 8A). The single mutants *nhr-66* and *nhr-80* also had reduced basal oxygen consumption rates of 6.12 and 7.44 pmoles/min/worm, respectively. Interestingly, the respiration rate for the *nhr-66; nhr-80* double mutant was lower than that of *nhr-49* with a rate of 3.87 pmoles/min/worm but higher than the electron transport chain (ETC) complex I defective mutant *gas-1(fc21)*, which showed rates of 2.55 pmoles/min/worm.

In a second assay for mitochondrial function, we indirectly measured β-oxidation by feeding the animals radiolabeled palmitic acid and then measuring the rates of production of an acid soluble metabolites, a byproduct of lipid oxidation. As the mitochondrial β-oxidation gene *acs-2* is down regulated in *nhr-49* mutants and contributes to their increased fat content, we used the *acs-2(ok2457)* mutant as a control [16]. Just like the *acs-2* mutants, the *nhr-49* animals also had a significant reduction in acid soluble metabolite production with a rate of 0.56 pmole/min/µg protein compared to 1.29 observed in wild-type animals (Figure 8B). Surprisingly, although the qRT-PCR analysis did not predict NHR-66 and NHR-80 to regulate genes in the β-oxidation pathway, the *nhr-66* and *nhr-80* single mutants also exhibited reduced oxidation rates (0.69 and 0.43 pmole/min/µg protein, respectively). This is consistent with our EM data that *nhr-66* and *nhr-80* mitochondria differ from wild-type, which may be explained by the reduced β-oxidation in these mutants. The β-oxidation rate for the *nhr-66; nhr-80* double mutants was also significantly reduced relative to wild-type animals (0.43 pmole/min/ug protein), although the rate in the double mutant was identical to the rate in the single mutant with the stronger effect (*nhr-80*), suggesting that the two NRs may act in the same pathway. Together, our data show that altered mitochondrial morphology strongly correlates with aberrant mitochondrial physiology, and they further suggest that several NRs play a significant role in maintaining normal mitochondrial function.

## Discussion

NHR-49 regulates genes involved in fatty acid β-oxidation and fatty acid desaturation [16], but its full influence on lipid metabolism remained obscure. Here, we reveal a previously uncharacterized group of NHR-49 targets, namely genes involved in the repression of sphingolipid processing and lipid remodeling. We also identify several NHR-49 interacting partner receptors and show that at least two of these, NHR-66 and NHR-80 directly bind to NHR-49 and modulate separable NHR-49 dependent pathways. In characterizing NHR-49's transcriptional network and studying its influence on physiology, our findings inform us on the evolutionary history of HNF4-related receptors across species and on their conserved metabolic pathways and physiological functions.

### NHR-49's transcriptional network

HNF4α plays an important role in mammalian physiology and despite the identification of several HNF4 bound and regulated genes, it is still unclear what the relevant targets are *in vivo*. The number of genes estimated to be regulated by HNF4α varies greatly and depends on the experimental approaches [33], [34]. For example, Odom *et al.* performed ChIP on chip to identify promoters occupied by HNF4α in the human liver and pancreas and identified 1575 potential HNF4 target genes [35]. In contrast, gene expression analysis of pancreatic cells in an HNF4α conditional knockout model identified only 133 genes as HNF4α regulated [36]. This highlighted that revealing the regulatory targets and processes of metabolic nuclear receptors needed further characterization. We therefore performed genome-wide transcriptional profiling to identify the targets of the PPAR/HNF4 like nuclear receptor NHR-49 in *C. elegans*. Our analysis revealed a previously unrecognized role of NHR-49 in the regulation of genes involved in membrane lipid metabolism, particularly glycosphingolipid processing. It will be important to determine whether mammalian HNF4α also regulates these genes.

**Figure 9:**
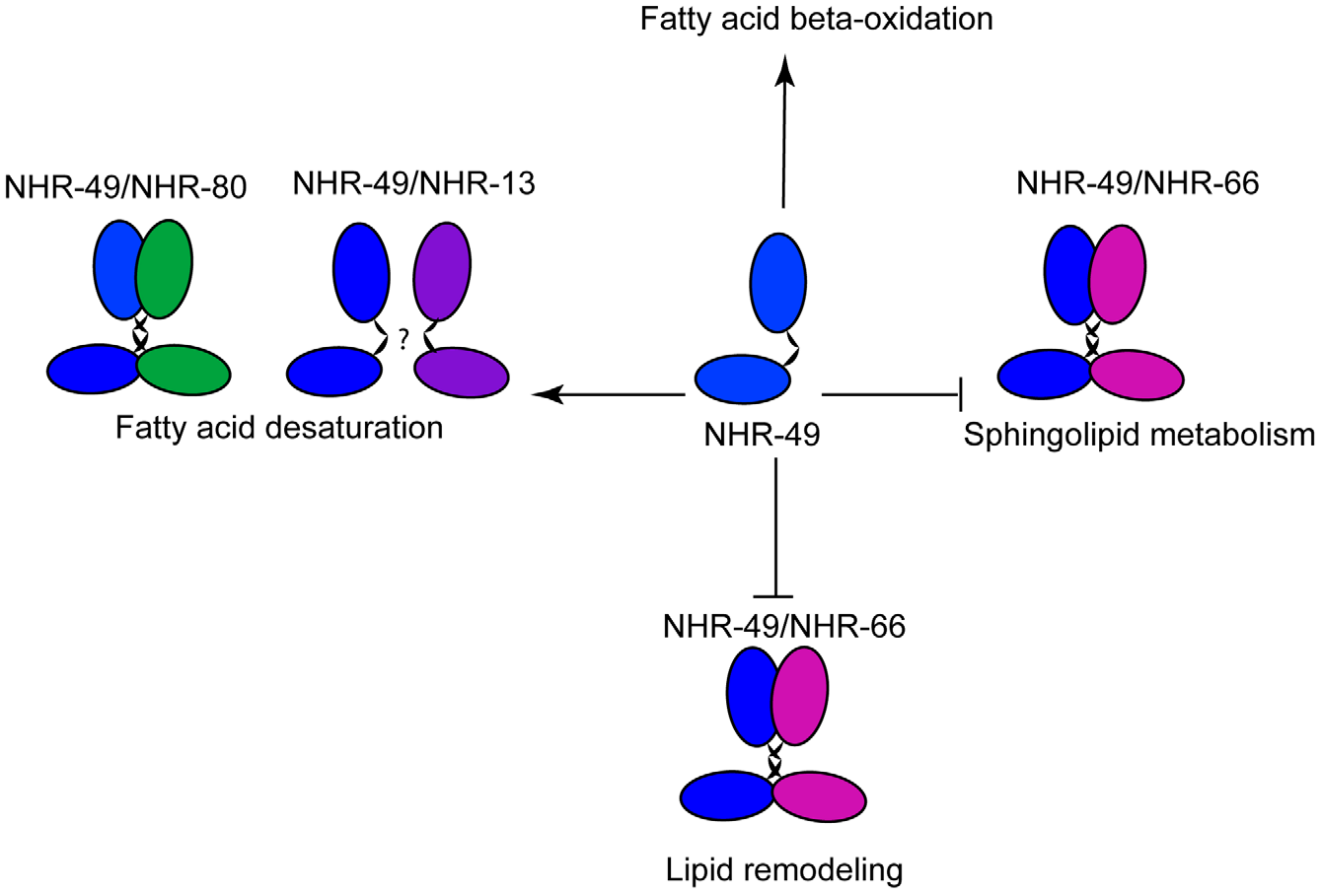
Model of NHR-49–dependent regulation of lipid metabolism. We propose that NHR-49 (blue) interacts with NHR-66 (pink) to repress genes involved in sphingolipid processing and lipid remodeling, whereas NHR-49 (blue) binds to NHR-80 (green) to activate the fatty acid desaturase genes.

It has also been unclear what co-factors of mammalian HNF4α influence distinct aspects of gene expression. We took advantage of *C. elegans* genetics to identify multiple partners of NHR-49 and confirmed that NHR-66 and NHR-80 physically interact with NHR-49 using *in vitro* binding assays. We further characterized the transcript level changes observed for genes regulated by NHR-66, NHR-80 and NHR-13. Notably, knockout of *nhr-66* affected only a subset of NHR-49 targets, sphingolipid and lipid remodeling target genes, whereas knockout of *nhr-80* and *nhr-13* affected the expression of NHR-49-regulated fatty acid desaturases. We therefore propose that NHR-49 and NHR-66 heterodimerize to regulate genes that mediate the breakdown of glycolipids and remodeling of lipid membranes, whereas NHR-49 cooperates with NHR-80 and possibly NHR-13 to sense and regulate the balance between saturated and unsaturated fat (Figure 9). Even though we did not detect a direct interaction between NHR-13 and NHR-49, NHR-13 clearly affects fatty acid desaturation. In the future, it will be important to determine if the different target genes are direct NR targets *in vivo*. To date, it has been difficult to identify a consensus binding sequence for NHR-49 and other NRs given the degeneracy of response elements often observed in *C. elegans*, and the lack of antibodies suitable for chromatin immunoprecipitation to define *in vivo* NR binding sites. GFP reporters of NHR-49/NHR-66 and NHR-49/NHR-80 regulated genes will be useful to determine whether these distinct dimers may selectively drive gene expression in individual tissues. Although HNF4 is not yet known to dimerize with other partners, it will be critical to determine if there are similar cofactors and/or it carries out similar mechanistic roles.

Another interesting finding of this study is the complexity of the NHR-49 regulatory network. For instance, both NHR-66 and NHR-80 are NHR-49 co-factors, but there are nuances in their co- regulation of NHR-49 genes. In the case of NHR-66, the fold changes observed in sphingolipid and lipid remodeling genes were comparable to those seen in *nhr-49* animals. However, in the case of NHR-80 and NHR-13, the levels of activation in fatty acid desaturation regulation did not completely parallel the levels we observed in *nhr-49* mutants. This suggests that additional regulation of these genes may involve NHR-49 hetero and/or homodimers or NHR-49 independent mechanisms, consistent with our observation that NHR-13 influences NHR-49 regulated desaturases but may not directly bind to NHR-49. Adding to the complexity, our gene expression data suggest that, in addition to the genes jointly regulated by NHR-49/NHR-66 and NHR-49/NHR-80, NHR-66 and NHR-80 regulate additional genes independently of NHR-49. In fact, our studies on mitochondrial morphology and function do not clearly differentiate between the possibilities that the observed defects are the result of NHR-49-dependent pathways or the result of multiple independent pathways regulated by NHR-66 and NHR-80. Our data also does not exclude the possibility that NHR-66 and NHR-80 act together for some functions. Future work will be needed to address these different scenarios and elucidate the complexity of this transcriptional network.

### Influence of NHR-49's target genes on physiology

Our lifespan analysis revealed that NHR-80 and NHR-13 regulated pathways appear to contribute to the early death of *nhr-49* deletion mutants. We propose that NHR-49's interaction with NHR-80 and possibly with NHR-13 modulates the conversion of saturated to unsaturated fat, and contributes to the shortened lifespan phenotype of *nhr-49* mutants. Supporting this idea, there is a significant correlation between the C18:0/C18:1n9 ratio and mean lifespan. This fits well with a recent study that showed the correlation between several fatty acid metabolic parameters to longevity, including the ratio of C18 to C18:1n9 [37]. Our data my also provide insight into the mechanism by which mammalian HNF4α protects against diabetes. Both *in vitro* and *in vivo* studies showed that pancreatic β-cells are highly susceptible to saturated fat-induced apoptosis or lipotoxicity [38], [39], whereas overexpression of the *fat-6*/*fat-7* homologue Stearoyl CoA Desaturase (SCD) protects against this lipotoxicity [40], [41]. It is intriguing to speculate that the shortened lifespan could be related to roles of HNF4 in protection against premature cell death.

Although NHR-49 is derived from the ancestral HNF4-like receptors [42], NHR-49 functionally resembles the PPARα. Our EM data suggest that mitochondrial morphology was abnormal in *nhr-49* mutants, which led us to test mitochondrial function in these worms. We observed defects in oxygen consumption and fatty acid β-oxidation, which may be the result of altered membrane lipids affecting the function of intestinal mitochondria. Because the co-factor mutants also exhibit mitochondrial defects, we propose that multiple pathways regulate mitochondrial function.

It has been shown that PPARs regulate the density (number) of mitochondria. In fact, PPARγ and PPARδ promote mitochondrial biogenesis in a cell-type specific manner, specifically increasing biogenesis in white adipose tissue and skeletal muscle, respectively [31], [32]. PPARs have also been shown to influence mitochondrial function by mostly regulating genes encoding mitochondrial fatty acid β -oxidation enzymes and mitochondrial uncouplers [43]. Our data suggest that *nhr-49* mutants differ from wild-type animals in the shape but not the fractional area (number) of their intestinal mitochondria. Given that some mitochondria in the *nhr-49* mutants are irregular in shape, it would be interesting to determine if the mitochondria in mammalian PPAR mutant mice exhibit similar phenotypes. Alternatively, a set of genes not commonly regulated by NHR-49 and PPAR may contribute to the altered morphology of the mitochondria in *nhr-49* mutants only. In line with this notion, our study revealed novel NHR-49 dependent targets and pathways, particularly glycosphingolipid processing and lipid remodeling, which mammalian PPARs are not known to regulate.

### Insights on evolution of the HNF4 family

When HNF4 and HNF4-like NRs are compared across species (Table 3), a common theme that emerges is the conservation of their roles in lipid metabolism pathways. It is possible that one ancestral role of HNF4–the mobilization of stored energy during nutrient deprivation and the regulation of β-oxidation–was adopted by PPARα, whereas other functions of HNF4 were either retained or outsourced to other NRs. In *C. elegans*, HNF4-like NRs underwent a massive expansion and it is likely that various functions were allocated to different nuclear receptors [44]. HNF4 paralogs in nematodes could then have been selected for divergent functions that included sphingolipid metabolism and lipid remodeling.

**Table 3:** Insights on the evolution of the HNF4 family across species.

Nuclear Receptors	Physiological Role	Metabolic Pathways	Structure Homo	Structure Hetero
MammalsHNF-4α	Liver differentiation, nutrient transport, lipid metabolism, xenobiotic metabolism, glucose metabolism, amino acid metabolism, blood maintenance, immune function		X [56]	
PPAR-α/RXR	Lipid metabolism, cholesterol metabolism, glucose metabolism, fasting response	Beta-oxidation, gluconeogenesis		X [57]
DrosophiladHNF-4	Lipid transport, lipid metabolism, fasting response, gut differentiation	Beta-oxidation, gluconeogenesis	X	
C.elegansNHR-49	Lipid metabolism, xenobiotic metabolism, fasting response	Beta-oxidation, fatty acid desaturation, sphingolipid processing, lipid remodeling, P450, gluconeogenesis	X	X
NHR-66	Lipid metabolism	Sphingolipid processing, lipid remodeling	?	X
NHR-80	Lipid metabolism	Fatty acid desaturation	?	X
NHR-13	Lipid metabolism	Fatty acid desaturation	?	X
YeastOAF-1/PIP2	Lipid metabolism		X [58]	X [59]

See text for details.

Mammalian RXR serves as a heterodimer partner for numerous NRs, including PPARs [45]. We thus speculate that like PPAR and its heterodimeric partner, RXR adopted specific HNF4 roles in mammals. It is possible that NHR-49 heterodimerized with other binding partners to adopt individual tasks that encompassed the numerous functions of the precursor nematode HNF4. This is particularly intriguing given the striking absence of any *C. elegans* orthologs of RXR-like molecules that in other organisms heterodimerize with a range of interacting proteins. NHR-49's action is promiscuous as it is clearly assuming different function with different partners. NHR-49 may therefore serve as an ancestral binding partner reminiscent of the mammalian RXR. *C. elegans* thus represents a class of animals that switched from solely using HNF4 receptors to regulate lipid metabolism to those that began to employ different heterodimeric partners to regulate distinct metabolic pathways.

In closing, our study has identified distinct binding partners that modulate specific pathways of NHR-49 activity. Additionally, we have also shown that these NRs play a role in mitochondrial physiology and function. Given the conservation of HNF4 structure, function and regulatory modules across species, it is possible that mammalian HNF4α utilizes similar mechanisms as that of *C. elegans* NHR-49, thus informing us of ways to selectively modulate its activity. Exploring these possibilities can assist in pharmacological efforts to manipulate mammalian HNF4α for specific desired outcomes without any detrimental side-effects.

## Materials and Methods

### Nematode strains and growth conditions

*C. elegans* strains N2 Bristol (wild-type), *nhr-49(nr2041)*, *nhr-66(ok940)*, *nhr-80(tm1011)*, *nhr-13(gk796)*, *gas-1(fc21)* and *acs-2*(*ok2457*) were grown at 20°C on high-growth plates seeded with OP50 bacteria and maintained as described [46]. The mutant strains were obtained from the CGC and were outcrossed at least five times with wild-type N2 worms. Single-worm PCR was used to determine the genotype of worms during crossing to generate *nhr-80; nhr-13* and *nhr-66; nhr-80* double-mutant lines. For mRNA and GC/MS analysis, worm embryos were allowed to hatch on unseeded nematode growth media (NGM)-lite plates overnight at 20°C. The next day, synchronized L1 larvae were plated onto NGM-lite plates seeded with *Escherichia coli* strain OP50. Worms were grown to early L4s at 20°C, harvested, washed three times with M9, and flash-frozen in liquid N_2_.

### Preparation of total nematode mRNA

*C. elegans* strains N2-Bristol (wild-type), *nhr-49(nr2041)*, *nhr-66(ok940)*, *nhr-80(tm1011)* and *nhr-13(gk796)* were grown at 20°C on high-growth plates seeded with OP50 bacteria and maintained as described [46]. Gravid adults from 10 10-cm plates were bleached, and embryos were dispersed onto 15-cm nematode growth media (NGM)-lite plates seeded with OP50. Worms at L4 stage were harvested, washed twice with M9, and frozen in liquid nitrogen. For RNA preparation, worms were thawed at 65°C for 10 min, and RNA was isolated using the Tri-Reagent Kit (Molecular Research Center, Cincinnati, Ohio, United States). Isolated total RNA was subjected to DNAase treatment and further purification using RNAeasy (Qiagen, Valencia, California).

### Yeast two-hybrid screen

For the NHR-49-LBD yeast two-hybrid screen [23], the strain AH109/pGBK-Leu2-NHR-49-LBD was used to probe a *C. elegans* oligo(dT)-primed cDNA library cloned into pPC86 (kindly provided by the Vidal laboratory, Dana Farber Cancer Institute, Boston). In short, ∼4.3×10^6^ independent transformants were screened for growth on medium-stringency plates (lacking tryptophan, leucine, histidine, and uracil containing 7.5 mM 3-amino-1,2,4-triazole (3-AT; Sigma, H-8056). Candidate clones were retested twice for their ability to grow on high-stringency medium (i.e. medium-stringency plates additionally lacking adenine), allowing recovery of 141 candidates. Following PCR and AluI digestion to identify redundant clones, 49 candidate plasmids were extracted and sequenced, yielding 24 independent cDNAs of 13 genes (Table S1c). To estimate the relative interaction strength of these candidate proteins with the NHR-49-LBD, plasmid pairs were transformed into strain Y187 (Clontech) and liquid β-galactosidase assays were performed as described in the manufacturer's protocol.

### qRT–PCR and microarray analysis

cDNA was prepared from 5 µg of total RNA in a 100-µl reaction using the Protoscript cDNA preparation kit (New England Biolabs, Beverly, Massachusetts, United States). Primer pairs were diluted into 96-well cell culture plates at a concentration of 3 µM. Next, 30-µl PCR reactions were prepared in 96-well plates. Each PCR reaction was carried out with TaqDNA Polymerase (Invitrogen, Carlsbad, California, United States) and consisted of the following reaction mixture: 0.3 µM primers, 1/500th of the cDNA reaction (corresponds to cDNA derived from 10 ng of total RNA), 125 µM dNTPs, 1.5 mM MgCl_2_, and 1× reaction buffer (20 mM Tris pH 8.4, 50 mM KCl), 0.15 µl (0.75 units) of TaqDNA Polymerase was used for each reaction. Formation of double-stranded DNA product was monitored using SYBR-Green (Molecular Probes, Eugene, Oregon, United States). Data were collected using RNA from at least three independent *C. elegans* growths. To determine the relationship between mRNA abundance and PCR cycle number, all primer sets were calibrated using serial dilutions of cDNA preparations. qRT-PCR primers were designed using Primer3 software [47]. qPCR was performed using a BioRad iCycler (MyiQ Single color).

For microarray analysis, total RNA was assayed for quality using an UV-Vis spectrophotometer and an Agilent 2100 Bioanalyzer (Agilent Technologies, Inc., Santa Clara, CA). Cy5- and Cy3-labeled cDNA targets were generated from 30 µg of total RNA using a reverse transcription, amino-allyl based labeling protocol [48]. Mutant and wild-type targets were co-hybridized to Washington University/Genome Sequencing Center *C. elegans* 23K spotted oligo arrays for 16 hours at 42°C, followed by a series of stringency washings at 42°C for 5 cycles in 2× SSC and 3 cycles in 0.1× SSC using an automated GeneTac Hybridization Station (Genomic Solutions, Ann Arbor, MI, USA). Post-hybridized arrays were scanned using a GenePix 4000B scanner (Axon Instruments, Union City, CA) and image analysis was performed using GenePix Pro software. Independent datasets were generated for each mutant *vs.* wild-type comparison: *nhr-49* (n = 2, with dye swap), *nhr-66* (n = 3), *nhr-80* (n = 4).

Array data was pre-processed through a custom-built application used to filter data based on criteria associated with spot-level signal quality (e.g., signal-to-noise, lower bound intensity threshold, spot dimension). Each array was background subtracted and the ratios (mutant/wt) were log_2_ transformed. Intra-array normalization was performed using a loess algorithm to correct for intensity-dependent ratio biasing [49]. For each comparison, the dataset was filtered by applying a lower-bound signal intensity cutoff, followed by the application of a variance filter using the ‘shorth’ function in the Bioconductor package *genefilter*. Pair-wise significance testing (mutant *vs.* wild-type) was performed using the Bioconductor package *limma* [20] and p-values were initially corrected for multiple testing using the false discovery rate (FDR) method of Benjamini and Hochberg [50]. We attempted to define differential expression as |log_2_(ratio)|≥0.848 with the FDR set to 5%, however the multiple testing penalty was too high to produce a gene list in some cases and as such we slightly relaxed our criteria by employing |log_2_(ratio)|≥0.848 and p-value≤0.001.

Gene ontology (GO) enrichment analysis was performed using GOrilla [21]. Each list from the *limma* analysis was ranked from smallest to largest p-value and analyzed for enriched biological process ontology terms found near the top of the list. Functional classification summary for the *nhr-49* mutant were presented as a scatter plot using the GO visualization tool REViGO [51].

### Fatty acid analysis

Fatty acids were isolated from 10,000 L4 animals grown on a single 15-cm NGM-Lite plate. Total lipids were extracted and converted to fatty acid methyl esters (FAMEs) as described [52]. After incubation at 80°C for 1 hour the samples were cooled and the FAMEs were extracted by adding water and hexane. FAMEs were analyzed for fatty acid composition by gas chromatography/mass spectrometry (GC/MS) (Agilent 5975GC, 6920MS). Peaks were assigned using fatty acid standards.

### Plasmids

The PCR products of the full length NHR-49, NHR-66, NHR-80 and NHR-13 were cloned into the expression vector pCS2^+^ that was generously provided by Dr. B. Eisenmann (Fred Hutchinson Cancer Research Center, Seattle). The PCR product for NHR-49 was flanked by BamH1/EcoR1, NHR-66 by Stu1/Xba1, NHR-80 by BamH1/EcoR1 and NHR-13 by BamH1/EcoR1 and then cloned into the corresponding sites in pCS2^+^. For bacterial expression, GST-NHR-49 was constructed by inserting BamH1-NHR-49 cDNA-EcoR1 into PGEX-2T vector. All clones were confirmed by sequence analysis. All plasmid requests should be directed to Stefan Taubert.

### GST–pull down assays

GST-fused to NHR-49 (GST-NHR-49) was induced with 1 mM IPTG for 2 hours at 23°C, expressed in the *Escherichia coli* BL21 (DE3) strain and purified using glutathione-Sepharose 4B beads. The IVTs were transcribed from the SP6 promoter and translated with the wheat germ system (Promega: Madison, WI), in the presence of unlabeled (Promega: Madison, WI) or ^35^S- labeled methionine (Perkin Elmer) according to the manufacturer's instructions. For the *in vitro* protein-protein interaction assays, the IVT protein was incubated with GST-NHR-49 for 90 minutes at 37°C and washed in binding buffer. The samples were then run on SDS-PAGE and the radiolabeled protein detected by autoradiography.

### Lifespan assays

Approximately 30–40 L1 worms were transferred to 6-cm plates seeded with L4440 RNAi bacteria and life-span assays were carried out at 20°C as described previously [53]. The L4 stage was counted as day 0. Adults were transferred to new plates daily until progeny production had ceased. Animals were considered dead when they no longer responded to a gentle tap with a worm pick. Life span curves and statistical data including p-values from Log-rank (Mantel-Cox) test were generated using GraphPad Prism version 5 software (GraphPad Software, San Diego, CA).

### High pressure transmission electron microscopy

Day 1 adults were placed into a 20% BSA/PBS buffer solution and prepared in a Leica-Impact-2 high-pressure freezer. In short, animals were kept for 60 hours in 100% acetone and uranyl acetate at −90°C. The temperature was then ramped from −90°C to −25°C over the course of 32.5 hours. Next, samples were incubated at −25°C for 13 hours. Finally, the temperature was brought from −25°C to 27°C in a 13 hour temperature ramp. Serial sections were post-stained in uranyl acetate followed by lead citrate. Thin cross sections were taken from resin-embedded clusters of young adults. Sections for adult animals were obtained from 3 different animals.

### Quantification of mitochondria

Ten micron transverse sections typically spanning the length of the worm from the pharynx to the vulva were examined per animal. Mitochondria from each section were individually outlined and the average measurements were obtained using Image J software. At least two animals were examined per mutant strain. Statistical data including p-values from unpaired t-tests using Welch's correction for unequal variance were generated using GraphPad Prism version 5 (GraphPad Software). Irregularity in mitochondrial shape was measured by counting the number of times the mitochondrial outer membrane changed from convex to concave. A turn was measured every time the mitochondria changed from convex to concave and vice versa.

### Determination of β-oxidation by acid-soluble metabolite production

The production of acid-soluble metabolites was used as an index of the β-oxidation of fatty acids based on an assay that was originally developed for cell lines [54]. We further modified the protocol for *C. elegans* based on the protocol reported by Mullaney *et al*, 2010 [55]. Synchronized L4 animals were washed off plates with 1× M9 medium and counted. The animals were then rinsed three times in 0.9% NaCl. An aliquot was stored at −80 C for subsequent protein determination. The worms were resuspended in S-Basal medium with ^14^C-palmitic acid (40–60 mCi/mmol) to a final concentration of 1 µCi /ml complexed to 25% fatty-acid-free BSA (all from GE Healthcare Life Sciences) per well. Samples were incubated on an orbital shaker for 2 hours at room temperature. Subsequently, 70% perchloric acid was added to precipitate the BSA-bound fatty acid. The samples were centrifuged for 10 minutes at 14,000 g and the radioactivity of the supernatant was determined by liquid scintillation. Samples without animals were included as background controls. These experiments were performed in triplicates two independent times. Statistical data was analyzed using one-way ANOVA and p-values were generated using GraphPad Prism version 5 (GraphPad Software).

### Measuring oxygen consumption rates by Seahorse XF-24 analyzer

Between 50 and 100 synchronized L4 worms were washed with 1XM9 and seeded into triplicates of the Seahorse XF-24 cell culture plates (Seahorse Bioscience, North Billerica, MA) in M9. Oxygen consumption rates were measured at least five times using the Seahorse XF-24 Analyzer (Seahorse Bioscience). Measurements were taken under basal conditions and were normalized to the number of worms counted per well. The Seahorse software was used to plot the results. The experiment was repeated two times under these conditions. Statistical data including p-values from unpaired t-test were generated using GraphPad Prism version 5 (GraphPad Software).

## Supporting Information

Table S1 Summary of gene expression data using qRT-PCR on *nhr-49*, *nhr-66*, *nhr-80* and *nhr-13* animals with respect to wild-type controls. Fold change is indicated in bold where expression levels are up regulated compared to wild-type.
(DOC)

Table S2 Summary of yeast-two-hybrid analysis (a) List of candidate NHR-49 interacting proteins identified in yeast two-hybrid screens using NHR-49-LBD (this study) or full-length NHR-49 [24] as bait, (b) Binding strengths of “preys” to the GAL4-DBD-NHR-49-LBD, (c) Information on Gal4-AD fusions.
(DOC)

Table S3 List of forward primers used for qRT-PCR.
(DOC)

Table S4 List of reverse primers used for qRT-PCR.
(DOC)

Table S5 List of differentially expressed genes in *nhr-66* compared to wild-type animals using microarray analysis. The data represent the analysis from three independent mRNA isolations and microarray hybridizations. “ID” refers to the identity of individual spots on the arrays, “logFC” represents the log of the fold change, “AveExpr” represents the averaged spot intensity.
(DOC)

Table S6 Occurrence of gene families in microarray results for NHR-66, based on GO terms.
(DOC)

Table S7 List of differentially expressed genes in *nhr-80* compared to wild-type animals using microarray analysis. The data represent the analysis from four independent mRNA isolations and microarray hybridizations. “ID” refers to the identity of individual spots on the arrays, “logFC” represents the log of the fold change, “AveExpr” represents the averaged spot intensity.
(DOC)

Table S8 Occurrence of gene families in microarray results for NHR-80, based on GO terms.
(DOC)

Table S9 List of common genes regulated by NHR-49 and NHR-66 and list of common genes regulated by NHR-49 and NHR-80 (a) Genes common to NHR-49 and NHR-66 (b) Genes common to NHR-49 and NHR-80.
(DOC)

Table S10 Summary of lifespan data.
(DOC)

Table S11 Lifespan and relative C18:0 fatty acid abundance.
(DOC)

## References

[1] Evans RM, Barish GD, Wang YX (2004). PPARs and the complex journey to obesity.. Nat Med.

[2] Chawla A, Repa JJ, Evans RM, Mangelsdorf DJ (2001). Nuclear receptors and lipid physiology: opening the X-files.. Science.

[3] Glass CK, Rosenfeld MG (2000). The coregulator exchange in transcriptional functions of nuclear receptors.. Genes & Development.

[4] Stoffel M, Duncan SA (1997). The maturity-onset diabetes of the young (MODY1) transcription factor HNF4α regulates expression of genes required for glucose transport and metabolism.. Proceedings of the National Academy of Sciences.

[5] Miquerol L, Lopez S, Cartier N, Tulliez M, Raymondjean M (1994). Expression of the L-type pyruvate kinase gene and the hepatocyte nuclear factor 4 transcription factor in exocrine and endocrine pancreas.. Journal of Biological Chemistry.

[6] Yamagata K, Furuta H, Oda N, Kaisaki PJ, Menzel S (1996). Mutations in the hepatocyte nuclear factor-4[alpha] gene in maturity-onset diabetes of the young (MODY1).. Nature.

[7] Hani EH, Suaud L, Boutin P, Chèvre JC, Durand E (1998). A missense mutation in hepatocyte nuclear factor-4 alpha, resulting in a reduced transactivation activity, in human late-onset non-insulin-dependent diabetes mellitus.. The Journal of Clinical Investigation.

[8] Love-Gregory LD, Wasson J, Ma J, Jin CH, Glaser B (2004). A Common Polymorphism in the Upstream Promoter Region of the Hepatocyte Nuclear Factor-4α Gene on Chromosome 20q Is Associated With Type 2 Diabetes and Appears to Contribute to the Evidence for Linkage in an Ashkenazi Jewish Population.. Diabetes.

[9] Silander K, Mohlke KL, Scott LJ, Peck EC, Hollstein P (2004). Genetic Variation Near the Hepatocyte Nuclear Factor-4α Gene Predicts Susceptibility to Type 2 Diabetes.. Diabetes.

[10] Gupta RK, Gao N, Gorski RK, White P, Hardy OT (2007). Expansion of adult β-cell mass in response to increased metabolic demand is dependent on HNF-4α.. Genes & Development.

[11] Rhodes CJ (2005). Type 2 Diabetes-a Matter of {beta}-Cell Life and Death?. Science.

[12] Hayhurst GP, Lee Y-H, Lambert G, Ward JM, Gonzalez FJ (2001). Hepatocyte Nuclear Factor 4{alpha} (Nuclear Receptor 2A1) Is Essential for Maintenance of Hepatic Gene Expression and Lipid Homeostasis.. Molecular and Cellular Biology.

[13] Weissglas-Volkov D, Huertas-Vazquez A, Suviolahti E, Lee J, Plaisier C (2006). Common Hepatic Nuclear Factor-4α Variants Are Associated With High Serum Lipid Levels and the Metabolic Syndrome.. Diabetes.

[14] Sluder AE, Maina CV (2001). Nuclear receptors in nematodes: themes and variations.. Trends in Genetics.

[15] Bertrand S, Brunet FG, Escriva H, Parmentier G, Laudet V (2004). Evolutionary Genomics of Nuclear Receptors: From Twenty-Five Ancestral Genes to Derived Endocrine Systems.. Molecular Biology and Evolution.

[16] Van Gilst MR, Hadjivassiliou H, Jolly A, Yamamoto KR (2005). Nuclear hormone receptor NHR-49 controls fat consumption and fatty acid composition in C. elegans.. PLoS Biol.

[17] Desvergne B, Wahli W (1999). Peroxisome Proliferator-Activated Receptors: Nuclear Control of Metabolism.. Endocr Rev.

[18] Wang Y-X, Lee C-H, Tiep S, Yu RT, Ham J (2003). Peroxisome-Proliferator-Activated Receptor [delta] Activates Fat Metabolism to Prevent Obesity.. Cell.

[19] Costet PLC, More J, Edgar A, Galtier P (1998). Peroxisome proliferator-activated receptor alpha-isoform deficiency leads to progressive dyslipidemia with sexually dimorphic obesity and statosis. .. J Biol Chem.

[20] Smyth GK (2005).

[21] Eden E, Navon R, Steinfeld I, Lipson D, Yakhini Z (2009). GOrilla: a tool for discovery and visualization of enriched GO terms in ranked gene lists.. BMC Bioinformatics.

[22] Aranda A, Pascual A (2001). Nuclear Hormone Receptors and Gene Expression.. Physiological Reviews.

[23] Taubert S, Van Gilst MR, Hansen M, Yamamoto KR (2006). A Mediator subunit, MDT-15, integrates regulation of fatty acid metabolism by NHR-49-dependent and -independent pathways in C. elegans.. Genes Dev.

[24] Li S, Armstrong CM, Bertin N, Ge H, Milstein S (2004). A Map of the Interactome Network of the Metazoan C. elegans.. Science.

[25] Brock TJ, Browse J, Watts JL (2006). Genetic regulation of unsaturated fatty acid composition in C. elegans.. PLoS Genet.

[26] Watts JL, Browse J (2000). A Palmitoyl-CoA-Specific Δ9 Fatty Acid Desaturase from Caenorhabditis elegans.. Biochemical and Biophysical Research Communications.

[27] Ntambi JM (1995). The regulation of stearoyl-CoA desaturase (SCD).. Progress in Lipid Research.

[28] Kniazeva M, Crawford QT, Seiber M, Wang C-Y, Han M (2004). Monomethyl Branched-Chain Fatty Acids Play an Essential Role in Caeonorhabditis elegans Development.. PLoS Biol.

[29] Ashrafi K, Chang FY, Watts JL, Fraser AG, Kamath RS (2003). Genome-wide RNAi analysis of Caenorhabditis elegans fat regulatory genes.. Nature.

[30] McKay RM, McKay JP, Avery L, Graff JM (2003). C. elegans: A Model for Exploring the Genetics of Fat Storage.. Developmental Cell.

[31] Bogacka I, Xie H, Bray GA, Smith SR (2005). Pioglitazone Induces Mitochondrial Biogenesis in Human Subcutaneous Adipose Tissue In Vivo.. Diabetes.

[32] Tanaka T, Yamamoto J, Iwasaki S, Asaba H, Hamura H (2003). Activation of peroxisome proliferator-activated receptor delta induces fatty acid beta-oxidation in skeletal muscle and attenuates metabolic syndrome.. Proc Natl Acad Sci U S A.

[33] Naiki T, Nagaki M, Shidoji Y, Kojima H, Imose M (2002). Analysis of Gene Expression Profile Induced by Hepatocyte Nuclear Factor 4α in Hepatoma Cells Using an Oligonucleotide Microarray.. Journal of Biological Chemistry.

[34] Lucas B, Grigo K, Erdmann S, Lausen J, Klein-Hitpass L (2005). HNF4[alpha] reduces proliferation of kidney cells and affects genes deregulated in renal cell carcinoma.. Oncogene.

[35] Odom DT, Zizlsperger N, Gordon DB, Bell GW, Rinaldi NJ (2004). Control of Pancreas and Liver Gene Expression by HNF Transcription Factors.. Science.

[36] Gupta RK, Gao N, Gorski RK, White P, Hardy OT (2007). Expansion of adult beta-cell mass in response to increased metabolic demand is dependent on HNF-4{alpha}.. Genes & Development.

[37] Shmookler Reis1, 3 RobertJ, Xu2 Lulu, Lee2 Hoonyong, Chae2,* Minho, Thaden2 JohnJ, Bharill1,3 Puneet, Tazearslan3,† Cagdas, Siegel4 Eric, Alla1 Ramani, Zimniak1,5 Piotr, Ayyadevara1,2 Srinivas (2011). Modulation of lipid biosynthesis contributes to stress resistance and longevity of C. elegans mutants.. Aging.

[38] Unger RH, Zhou YT (2001). Lipotoxicity of beta-cells in obesity and in other causes of fatty acid spillover.. Diabetes.

[39] Shimabukuro M, Higa M, Zhou Y-T, Wang M-Y, Newgard CB (1998). Lipoapoptosis in Beta-cells of Obese Prediabeticfa/fa Rats.. Journal of Biological Chemistry.

[40] Peter A, Weigert C, Staiger H, Rittig K, Cegan A (2008). Induction of stearoyl-CoA desaturase protects human arterial endothelial cells against lipotoxicity.. American Journal of Physiology - Endocrinology And Metabolism.

[41] Listenberger LL, Han X, Lewis SE, Cases S, Farese RV (2003). Triglyceride accumulation protects against fatty acid-induced lipotoxicity.. Proceedings of the National Academy of Sciences.

[42] Robinson-Rechavi M, Maina CV, Gissendanner CR, Laudet V, Sluder A (2005). Explosive Lineage-Specific Expansion of the Orphan Nuclear Receptor HNF4 in Nematodes.. Journal of Molecular Evolution.

[43] Puigserver P, Wu Z, Park CW, Graves R, Wright M (1998). A Cold-Inducible Coactivator of Nuclear Receptors Linked to Adaptive Thermogenesis.. Cell.

[44] Taubert S, Ward JD, Yamamoto KR (2011). Nuclear hormone receptors in nematodes: Evolution and function.. Molecular and Cellular Endocrinology.

[45] Mangelsdorf DJ, Evans RM (1995). The RXR heterodimers and orphan receptors.. Cell.

[46] Brenner S (1974). THE GENETICS OF CAENORHABDITIS ELEGANS.. Genetics.

[47] Skaletsky SRaHJ (2000). Primer3 on the www for general users and for biologist programmers.. Methods Mol Biol.

[48] Fazzio TG, Kooperberg C, Goldmark JP, Neal C, Basom R (2001). Widespread Collaboration of Isw2 and Sin3-Rpd3 Chromatin Remodeling Complexes in Transcriptional Repression.. Molecular and Cellular Biology.

[49] Yang YH, Dudoit S, Luu P, Lin DM, Peng V (2002). Normalization for cDNA microarray data: a robust composite method addressing single and multiple slide systematic variation.. Nucleic Acids Research.

[50] Benjaminini YaHY (1995). Controlling the false discovery rate: a practical and powerful approach to multiple testing.. J R Statistical Soc Ser B-Methodological.

[51] Supek F, Bošnjak M, Škunca N, Šmuc T (2011). REVIGO Summarizes and Visualizes Long Lists of Gene Ontology Terms.. PLoS ONE.

[52] Watts JL, Browse J (2002). Genetic dissection of polyunsaturated fatty acid synthesis in Caenorhabditis elegans.. Proc Natl Acad Sci U S A.

[53] Hansen M, Hsu A-L, Dillin A, Kenyon C (2005). New Genes Tied to Endocrine, Metabolic, and Dietary Regulation of Lifespan from a *Caenorhabditis elegans* Genomic RNAi Screen.. PLoS Genet.

[54] Golej DL, Askari B, Kramer F, Barnhart S, Vivekanandan-Giri A (2011). Long-chain acyl-CoA synthetase 4 modulates prostaglandin E2 release from human arterial smooth muscle cells.. Journal of Lipid Research.

[55] Mullaney BC, Blind RD, Lemieux GA, Perez CL, Elle IC (2010). Regulation of C. elegans Fat Uptake and Storage by Acyl-CoA Synthase-3 Is Dependent on NR5A Family Nuclear Hormone Receptor nhr-25.. Cell Metabolism.

[56] Jiang G, Nepomuceno L, Hopkins K, Sladek F (1995). Exclusive homodimerization of the orphan receptor hepatocyte nuclear factor 4 defines a new subclass of nuclear receptors.. Mol Cell Biol.

[57] Schulman IG, Shao G, Heyman RA (1998). Transactivation by Retinoid X Receptor-Peroxisome Proliferator-Activated Receptor gamma (PPARgamma ) Heterodimers: Intermolecular Synergy Requires Only the PPARgamma Hormone-Dependent Activation Function.. Mol Cell Biol.

[58] Trzcinska-Danielewicz J, Ishikawa T, Micialkiewicz A, Fronk J (2008). Yeast transcription factor Oaf1 forms homodimer and induces some oleate-responsive genes in absence of Pip2.. Biochemical and Biophysical Research Communications.

[59] Rottensteiner H, Kal AJ, Hamilton B, Ruis H, Tabak HF (1997). A Heterodimer of the Zn2Cys6 Transcription Factors Pip2p and Oaf1p Controls Induction of Genes Encoding Peroxisomal Proteins in Saccharomyces Cerevisiae.. European Journal of Biochemistry.

