# Supplementary material for: Coordinate Regulation of Lipid Metabolism by Novel Nuclear Receptor Partnerships
Source: PLoS Genet. 2012 Apr 12;8(4):e1002645. doi: 10.1371/journal.pgen.1002645 (PMC3325191; doi:10.1371/journal.pgen.1002645)
Supplement: Table S1 — Summary of gene expression data using qRT-PCR on nhr-49, nhr-66, nhr-80 and nhr-13 animals with respect to wild-type controls. Fold change is indicated in bold where expression levels are up regulated compared to wild-type. (DOC) [file pgen.1002645.s001.doc]

Table S1.

| Gene Name | WormBase sequence name | Overall fold change with respect to wild-type | | | |
| --- | --- | --- | --- | --- | --- |
|  |  | *nhr-49* | *nhr-66* | *nhr-80* | *nhr-13* |
| acid ceramidase | F27E5.1 | **2.29+/-0.7** | **2.29+/-0.5** | 1+/-0.6 | 1.7+/-0.5 |
| glycosyl hydrolase | E02H9.5 | **4+/-1.1** | **4+/-1.5** | 0.5+/-0.2 | 0.5+/-0.2 |
| sphingosine phosphate lyase | B0222.4 | **32+/-12.3** | **16+/-5.4** | 0.2+/-0.2 | 0.2+/-0.2 |
| phospholipase | Y65B4BR.1 | **8+/-3.4** | **4+/-2.2** | 0.2+/-0.2 | 0.4+/-0.4 |
| phospholipase | W02B12.1 | **2+/-0.5** | **2+/-0.3** | 0.3+/-0.1 | 0.4+/-0.1 |
| TAG ligase | ZK617.2 | **32+/-9.3** | **16+/-6.1** | 1+/-0.4 | 1.7+/-1 |
| O-acyltransferase | Y67A10A.1 | **4+/-1.36** | **8+/-2.7** | 0.5+/-0.6 | 1+/-0.7 |
| *fat-7* | F10D2.9 | 128+/-53 | 1+/-0.3 | 128+/-40 | 73.5+/-16.9 |
| *fat-5* | W06D12.3 | 16+/-2.4 | 1+/-0.1 | 8+/-5.9 | 2.14+/-0.8 |
| *fat-6* | VZK822L.1 | 2+/-0.4 | 1+/-0.3 | 4+/-1.2 | 1.5+/-0.3 |
| *acs-2* | F28F8.2 | 8+/-3.3 | 1+/-0.6 | 1+/-0.4 | 1.1+/-0.3 |
| *cpt-5* | F09F3.9 | 16+/-2.4 | 1+/-0.3 | 2+/-0.4 | 0.87+/-0.1 |
| *ech-1* | C29F3.1 | 2+/-0.4 | 1+/-0.2 | 2+/-1.0 | 3.24+/-0.5 |
