# Supplementary material for: Coordinate Regulation of Lipid Metabolism by Novel Nuclear Receptor Partnerships
Source: PLoS Genet. 2012 Apr 12;8(4):e1002645. doi: 10.1371/journal.pgen.1002645 (PMC3325191; doi:10.1371/journal.pgen.1002645)
Supplement: Table S2 — Summary of yeast-two-hybrid analysis (a) List of candidate NHR-49 interacting proteins identified in yeast two-hybrid screens using NHR-49-LBD (this study) or full-length NHR-49 [24] as bait, (b) Binding strengths of “preys” to the GAL4-DBD-NHR-49-LBD, (c) Information on Gal4-AD fusions. (DOC) [file pgen.1002645.s002.doc]

Table S2a.

| Prey gene name | Prey WormBase sequence name. | Bait  NHR-49-LBD | Bait  NHR-49 full-length |
| --- | --- | --- | --- |
| NHR-11 | ZC410.1 | ✔ |  |
| NHR-13 | Y5H2B.2 | ✔ | ✔ |
| NHR-19 | E02H1.7 |  | ✔ |
| NHR-22 | K06A1.4 | ✔ | ✔ |
| NHR-66 | T09A12.4 | ✔ | ✔ |
| NHR-49 | K10C3.6 |  | ✔ |
| NHR-71 | K11E4.5 |  | ✔ |
| NHR-73 | C27C7.4 |  | ✔ |
| NHR-76 | C05G6.1 |  | ✔ |
| NHR-79 | T26H2.9 | ✔ | ✔ |
| NHR-105 | C06G3.1 | ✔ | ✔ |
| NHR-234 | Y38E10A.18 |  | ✔ |
| NHR-243 | Y80D3A.4 |  | ✔ |
| NHR-247 | ZK1037.5 |  | ✔ |
| NHR-256 | ZK697.2 | ✔ | ✔ |
| MDT-15 | R12B2.5 | ✔ |  |
|  | F54D10.7 |  | ✔ |
| HMBX-1 | F54A5.1 |  | ✔ |
| SCD-1 | H20J18.1 |  | ✔ |
| TTR-27 | R90.2 |  | ✔ |

Table S2b.

|  | Avg Gal4-DBD | Avg NHR-49-LBD | SEM Gal4-DBD | SEM NHR-49-LBD |
| --- | --- | --- | --- | --- |
| NHR-11aa1 | 2 | 565 | 2.3 | 68.8 |
| NHR-13aa | -3 | 658 | 0.5 | 72.3 |
| NHR-22aa228 | 0 | 1070 | 0.7 | 92.2 |
| NHR-66aa182 | -1 | 457 | 0.9 | 29.1 |
| NHR-105aa1 | -1 | 727 | 0.6 | 66.1 |
| MDT-15aa(-33) | 3 | 613 | 1.2 | 85.0 |
| NHR-79aa1 | 2 | 12 | 1.5 | 1.7 |
| PQN-82 | 3 | 58 | 2.5 | 21.0 |
| RAD-51 | 2 | 32 | 1.2 | 2.6 |
| Filamin | 15 | 50 | 2.3 | 5.7 |
| DIM-1 | 8 | 5 | 2.8 | 2.1 |
| ATP-CL | 30 | 42 | 5.2 | 6.3 |
|  |  |  |  |  |
| n=6 |  |  |  |  |
|  |  |  |  |  |
|  |  |  |  |  |

Table S2c.

| Gene | Gal4-AD fusion starts at aa | In Frame | # of times cDNA fished (as determined by sequencing) |
| --- | --- | --- | --- |
| NHR-11 | 1 | Yes | 1 |
| NHR-13 | -2 (linker) | Yes | 11 |
| NHR-13 | 4 | Yes | 1 |
| NHR-13 | 72 | Yes | 1 |
| NHR-22 | -4 (linker) | Yes | 1 |
| NHR-22 | 1 | Yes | 1 |
| NHR-22 | 4 | Yes | 1 |
| NHR-22 | 15 | Yes | 1 |
| NHR-22 | 228 | Yes | 4 |
| NHR-66 | 201 | Yes | 1 |
| NHR-66 | 182 | Yes | 3 |
| NHR-66 | 198 | Yes | 1 |
| NHR-66 | 347 | Yes | 1 |
| NHR-79 | 1 | Yes | 1 |
| NHR-105 | -4 (linker) | Yes | 1 |
| NHR-105 | -2(linker) | Yes | 5 |
| NHR-256 | 2 | Yes | 5 |
| MDT-15 | -33 (linker) | Yes | 3 |
| PQN-82 | 18 | Yes | 1 |
| RAD-51 | 39 | Yes | 1 |
| DIM-1 | 480 | Yes | 1 |
| B0365.1 | 745 | Yes | 1 |
| Y66H1B.2 | 999 | Yes | 1 |
| Unidentifiable |  |  | 1 |
