# Supplementary material for: Coordinate Regulation of Lipid Metabolism by Novel Nuclear Receptor Partnerships
Source: PLoS Genet. 2012 Apr 12;8(4):e1002645. doi: 10.1371/journal.pgen.1002645 (PMC3325191; doi:10.1371/journal.pgen.1002645)
Supplement: Table S4 — List of reverse primers used for qRT-PCR. (DOC) [file pgen.1002645.s004.doc]

Table S4.

| Well | Oligo Name | Sequence (5' to 3') | Plate Name |
| --- | --- | --- | --- |
| A1 | B0348.2 | tggatgaatcacagcaaagc | plate 2 |
| A2 | B0222.4 | gaacaaccagcaggaagagg | plate 2 |
| A3 | F45E4.1 | ggcatatcctgcttgtttgc | plate 2 |
| A4 | R08H2.9 | agccacactcctttgattcc | plate 2 |
| A5 | Y65B4BR.1 | gaatctcctgctgagcatcc | plate 2 |
| A6 | Y37H2A.11 | atctcggtgctcacaattcc | plate 2 |
| A7 | C29F3.5 | atgtgaaggaactgggatgg | plate 2 |
| A8 | ZK218.5 | ctgcaacctccaatgattcc | plate 2 |
| A9 | W03B1.6 | acctcgcatgacaatgtgg | plate 2 |
| A10 | ZK617.2 | cttggaaagaccagcagtgg | plate 2 |
| A11 | F27E5.1 | cgaaaagctgtccaaagtcc | plate 2 |
| A12 | E02H9.5 | gccttcctcgttgatagtcg | plate 2 |
| B1 | T10B9.1 | cttttgcacttgcctcatcc | plate 2 |
| B2 | C36C5.14 | tcacacttgctgacgagtcc | plate 2 |
| B3 | C29F3.2 | attcaggcgagaaaacttcg | plate 2 |
| B4 | T24C4.4 | aataaccgggaagaacaaacc | plate 2 |
| B5 | F54F3.3 | ctttaagtccagcgcaaacc | plate 2 |
| B6 | ZK218.3 | gggcaggagtgtagaagtcg | plate 2 |
| B7 | Y46C8AL.4 | tggaggaagcacttgaaacc | plate 2 |
| B8 | C47F8.5 | ctcgatagtcctccgacacc | plate 2 |
| B9 | C47A10.1 | gatgtttcgggttcatttgg | plate 2 |
| B10 | C04G6.5 | tcctggaagccagaagagc | plate 2 |
| B11 | F35E8.8 | gccaaggagttgacttgagc | plate 2 |
| B12 | C47F8.8 | tatcaccttttgcgctttcc | plate 2 |
| C1 | T19D12.4 | tcacgaactccggttactcc | plate 2 |
| C2 | Y67A10A.1 | tggcgcaaatctacaataacc | plate 2 |
| C3 | F57B9.6 | gatcctgaacggaactctcg | plate 2 |
| C4 | ZK218.11 | tcacgcaattagtcgcttcc | plate 2 |
| C5 | C18A11.1 | caccatgcacacaaatctcc | plate 2 |
| C6 | Y73F8A.8 | agatggttgacattggactgg | plate 2 |
| C7 | F37B1.3 | tcttctcccaggtgtcatcc | plate 2 |
| C8 | T16G1.6 | tcgtcgttgaacatcgtacc | plate 2 |
| C9 | T24E12.5 | ttcgtggttgttgtctctcg | plate 2 |
| C10 | C54E4.5 | agtttgcctcatccacatcc | plate 2 |
| C11 | F45D11.1 | cgccataatcactttgagagg | plate 2 |
| C12 | C53B4.7b | tacaattcggatgtggatgc | plate 2 |
| D1 | R52.6 | ctcctaattccaatgtccatcc | plate 2 |
| D2 | W02B12.1 | atatttccatgcccgtaagc | plate 2 |
| D3 | ZC443.5 | ctccattgtactgcccaacc | plate 2 |
| D4 | K12G11.3 | tcctccctcagtcaatttgg | plate 2 |
| D5 | C45B11.3 | tttactgctgcctcatcacg | plate 2 |
| D6 | F18E3.7a | ccataggctctttgttgttgc | plate 2 |
| D7 | C32H11.12 | agctctgtccatcccatcc | plate 2 |
| D8 | Y19D10A.9 | tagaaacctgcggagactgg | plate 2 |
| D9 | Y38E10A.15 | gtggaacttgagcccatcc | plate 2 |
| D10 | T09F5.9 | ttagtcgaatctgcccaagc | plate 2 |
| D11 | F23C8.4 | atcctcggtgaacactttgc | plate 2 |
| D12 | Y75B8A.4 | tgcattctgattggagatgg | plate 2 |
| E1 | C47F8.1 | aggctctttttcacgagacg | plate 2 |
| E2 | R09D1.7 | gttgccccagttatcattcc | plate 2 |
| E3 | F48G7.3 | atttgctggatgtcgtagcc | plate 2 |
| E4 | F45C12.1 | tgatgttcaccgacacttgg | plate 2 |
| E5 | C53A3.2 | tctgcagtttggatcaagtcc | plate 2 |
| E6 | R13D11.1 | agagcaactgccatgaaacc | plate 2 |
| E7 | Y34F4.2 | agtttgccagatgcttttcg | plate 2 |
| E8 | C36A4.1 | gtgtcggatgtcaatgttcg | plate 2 |
| E9 | F53G2.1 | agcgttgaagaacactgtcg | plate 2 |
| E10 | F11A5.3 | ctgctccaagtgaacgaagg | plate 2 |
| E11 | R08C7.8 | gctcgtaagaatccgagacg | plate 2 |
| E12 | ZK265.1 | tttgcgacttcaactgattcc | plate 2 |
| F1 | Y73B6BL.11 | actgccaaatggatctctcg | plate 2 |
| F2 | M04C9.4 | tggcggagacttttcatagc | plate 2 |
| F3 | C55A1.6 | gcgataaacttgtcggaagc | plate 2 |
| F4 | T03D3.1 | tgtgtcgttcgccttatcc | plate 2 |
| F5 | C45E5.1 | gcggctttcatcagtttagg | plate 2 |
| F6 | K10C2.3 | aatcccaatcccaaaattcc | plate 2 |
| F7 | ZK550.6 | tgcgtagtgacaggagatcg | plate 2 |
| F8 | R106.2 | gagacgactgggcaaatag | plate 2 |
| F9 | T05B4.3 | gtgacagagtcgcattgagc | plate 2 |
| F10 | F19B10.2 | cataatctccgcatccttgc | plate 2 |
| F11 | C33F10.1 | gaggctttttggtgatttgg | plate 2 |
| F12 | K09H11.7 | gagatttggtggcattattgg | plate 2 |
| G1 | K10C3.6a | gttgtcgcattggccagac | plate 2 |
| G2 | fat-1 | tagagaattgtgagtgccgc | plate 2 |
| G3 | fat-2 | cattgagccattcataatcgg | plate 2 |
| G4 | Gei-7a | gacaactgatccggctcgg | plate 2 |
| G5 | ACS-2 | gggaagaccacagtttgtcc | plate 2 |
| G6 | NHR-23 | acttgtggcgatgggaagc | plate 2 |
| G7 | R09B5.6 | tttcctccaaggctgctgg | plate 2 |
| G8 | K11D12.4 | agagtggatatcttccagcc | plate 2 |
| G9 | Y48G9A.10 | cgcattggcaggtactactg | plate 2 |
| G10 | F09F3.9 | gtagagcatatcgccctgc | plate 2 |
| G11 | F40F4.3 | gtttacgagtgaaccatccg | plate 2 |
| G12 | T22G5.6 | ctctctaattttgaaagcgagc | plate 2 |
| H1 | E04F6.5b | acaatttcagcgactctggc | plate 2 |
| H2 | Fat-7 | caatgatgtcgttttgaagagc | plate 2 |
| H3 | Fat-5 | aacacaagctgatatagtccg | plate 2 |
| H4 | Fat-6 | gatgacgtcattttgaagagc | plate 2 |
| H5 | C29F3.1 | aattgtcctccactcttcgc | plate 2 |
| H6 | T05G5.6 | catctccttgatatcagctcc | plate 2 |
| H7 | C55B7.4A | gtgatgcaaacagtttcgcc | plate 2 |
| H8 | F28D1.9 | tgataaattggcatcgagacg | plate 2 |
| H9 | C02B10.1 | ctcttcagatccatttctcac | plate 2 |
| H10 | nhr-80 | ttttcaagctttgcctgacc | plate 2 |
| H11 | nhr-80b | acattgcagtcatcccttcc | plate 2 |
| H12 | nhr-66c | agttgaatccgtccagtctagc | plate 2 |
