# Supplementary material for: Coordinate Regulation of Lipid Metabolism by Novel Nuclear Receptor Partnerships
Source: PLoS Genet. 2012 Apr 12;8(4):e1002645. doi: 10.1371/journal.pgen.1002645 (PMC3325191; doi:10.1371/journal.pgen.1002645)
Supplement: Table S5 — List of differentially expressed genes in nhr-66 compared to wild-type animals using microarray analysis. The data represent the analysis from three independent mRNA isolations and microarray hybridizations. “ID” refers to the identity of individual spots on the arrays, “logFC” represents the log of the fold change, “AveExpr” represents the averaged spot intensity. (DOC) [file pgen.1002645.s005.doc]

Table S5.

| ID | Name | logFC | AveExpr | P.Value | Gene WB ID | Gene Public Name |
| --- | --- | --- | --- | --- | --- | --- |
| cea2.i.48948 | ZK218.5 | 7.11 | 7.4 | 1.82E-09 | WBGene00013939 | ZK218.5 |
| cea2.i.03172 | F56H6.5 | 5.59 | 7.27 | 4.70E-09 | WBGene00010166 | gmd-2 |
| cea2.i.31956 | B0222.4 | 5.27 | 7.86 | 3.22E-08 | WBGene00006418 | tag-38 |
| cea2.i.06753 | Y67A10A.1 | 3.19 | 8.33 | 4.18E-08 | WBGene00013450 | Y67A10A.1 |
| cea2.c.38150 | T05B4.3 | -5.9 | 7.29 | 6.04E-08 | WBGene00020237 | phat-4 |
| cea2.i.36289 | F11A5.5 | 2.45 | 7.23 | 4.40E-07 | WBGene00008673 | F11A5.5 |
| cea2.i.06720 | Y65B4BR.1 | 4.02 | 6.6 | 4.74E-07 | WBGene00022040 | Y65B4BR.1 |
| cea2.i.38647 | F38H12.3 | 2.22 | 7.4 | 8.31E-07 | WBGene00018189 | nhr-181 |
| cea2.p.158384 | F08F8.5 | 2.1 | 6.97 | 1.07E-06 | WBGene00017270 | numr-1 |
| cea2.i.32048 | B0348.2 | 3.29 | 8.59 | 1.49E-06 | WBGene00015152 | B0348.2 |
| cea2.i.30269 | Y46C8AL.2 | 2.44 | 7.34 | 1.59E-06 | WBGene00021580 | clec-174 |
| cea2.i.31736 | ZK617.2 | 3.97 | 9.27 | 2.79E-06 | WBGene00014009 | lips-6 |
| cea2.d.01787 | C53B4.7b | 2.85 | 6.85 | 3.37E-06 | NA | NA |
| cea2.i.27460 | K11H12.4 | 2.23 | 8.28 | 3.31E-06 | WBGene00019660 | K11H12.4 |
| cea2.c.38832 | T27E4.4 | -3.4 | 9.52 | 4.05E-06 | WBGene00020862 | fip-2 |
| cea2.i.32177 | B0554.4 | 2.57 | 6.32 | 5.39E-06 | WBGene00015257 | B0554.4 |
| cea2.i.11595 | F27E5.1 | 2.21 | 9.09 | 5.10E-06 | WBGene00009192 | F27E5.1 |
| cea2.i.02605 | F41D3.11 | 4.06 | 6.4 | 7.14E-06 | WBGene00009616 | F41D3.11 |
| cea2.d.29913 | F57E7.1 | 2.36 | 6.34 | 1.00E-05 | WBGene00010202 | F57E7.1 |
| cea2.i.08369 | B0454.8 | 2.03 | 7.42 | 1.71E-05 | WBGene00015199 | B0454.8 |
| cea2.p.47251 | T19D12.4 | 1.53 | 9.64 | 2.12E-05 | WBGene00020579 | T19D12.4 |
| cea2.d.48138 | ZK1025.5 | -2.3 | 6.66 | 2.84E-05 | WBGene00014185 | ZK1025.5 |
| cea2.i.32798 | C07G3.2 | 1.93 | 6.8 | 3.62E-05 | WBGene00015574 | C07G3.2 |
| cea2.p.48441 | W02B12.1 | 1.63 | 9.38 | 3.40E-05 | WBGene00012201 | W02B12.1 |
| cea2.d.48143 | ZK1025.6 | -1.5 | 7.68 | 3.53E-05 | WBGene00014186 | nhr-244 |
| cea2.p.115997 | F35E12.8 | 1.38 | 8.38 | 7.03E-05 | WBGene00009432 | F35E12.8 |
| cea2.d.00021 | B0218.8 | -1.3 | 8.57 | 8.02E-05 | WBGene00015052 | clec-52 |
| cea2.d.30172 | F58B3.2 | 2.07 | 9.3 | 8.45E-05 | WBGene00003094 | lys-5 |
| cea2.d.14571 | C27C7.8 | -1.7 | 5.61 | 9.75E-05 | WBGene00007770 | nhr-259 |
| cea2.p.38566 | F35C5.9 | 1.2 | 7.78 | 0.000102 | WBGene00009397 | clec-66 |
| cea2.p.127324 | W08G11.1 | 1.14 | 7.67 | 0.000117 | WBGene00012346 | W08G11.1 |
| cea2.i.07449 | ZK1025.3 | -3.9 | 7.75 | 0.000116 | WBGene00014183 | ZK1025.3 |
| cea2.c.49413 | R12E2.7 | 1.21 | 12.6 | 0.000127 | WBGene00020033 | R12E2.7 |
| cea2.d.23620 | F35D11.11b | -1.2 | 8.05 | 0.000135 | NA | NA |
| cea2.p.93077 | F49C12.7 | 1.33 | 8.43 | 0.000144 | WBGene00009877 | F49C12.7 |
| cea2.c.09458 | C04G6.5 | 1.07 | 8.59 | 0.000151 | WBGene00015455 | C04G6.5 |
| cea2.p.112433 | F10A3.4 | 1.35 | 10 | 0.000179 | WBGene00008634 | F10A3.4 |
| cea2.p.157756 | ZK678.5 | 1.19 | 10.1 | 0.000193 | WBGene00006950 | wrt-4 |
| cea2.d.65625 | R12E2.15 | 1.08 | 13.4 | 0.000202 | WBGene00020040 | R12E2.15 |
| cea2.d.41903 | Y105C5A.4 | 1.61 | 9.59 | 0.000218 | WBGene00000028 | abu-5 |
| cea2.d.47046 | Y73F8A.9 | 1.86 | 9.39 | 0.000237 | WBGene00004171 | pqn-91 |
| cea2.i.33937 | C29F3.5 | -2.3 | 6.78 | 0.000277 | WBGene00007806 | clec-230 |
| cea2.p.110949 | C47A10.1 | 1.04 | 9.2 | 0.000265 | WBGene00004003 | pgp-9 |
| cea2.p.158399 | R12E2.14 | 1.52 | 11.6 | 0.000272 | WBGene00020039 | R12E2.14 |
| cea2.d.39379 | T20D4.7 | 1.96 | 6.27 | 0.000297 | WBGene00020613 | T20D4.7 |
| cea2.p.38384 | F33A8.7 | 1.22 | 7.58 | 0.000311 | WBGene00009355 | F33A8.7 |
| cea2.d.05343 | T05H4.4 | 1.39 | 6.31 | 0.000307 | WBGene00020267 | T05H4.4 |
| cea2.i.30277 | Y46C8AL.4 | 3.43 | 6.26 | 0.000319 | WBGene00021582 | clec-71 |
| cea2.d.04924 | R09B5.3 | 0.97 | 7.14 | 0.000332 | WBGene00000556 | cnc-2 |
| cea2.3.07963 | E02H9.5 | 1.81 | 6.11 | 0.000397 | WBGene00017103 | E02H9.5 |
| cea2.i.12394 | F43C11.3 | 1.13 | 7.85 | 0.000395 | WBGene00018380 | F43C11.3 |
| cea2.i.54612 | F54B11.11 | 0.94 | 9.34 | 0.000422 | WBGene00010034 | F54B11.11 |
| cea2.d.14568 | C27C7.4 | -1.9 | 5.87 | 0.000437 | WBGene00003663 | nhr-73 |
| cea2.d.47041 | Y73F8A.9 | 1.67 | 8.08 | 0.000445 | WBGene00004171 | pqn-91 |
| cea2.p.90496 | F13E9.8 | 1.69 | 6.94 | 0.000555 | WBGene00008757 | F13E9.8 |
| cea2.c.06773 | W05F2.3 | -1.3 | 8.78 | 0.000549 | WBGene00021035 | W05F2.3 |
| cea2.i.59795 | Y73B6BL.15 | 1.16 | 11.3 | 0.000627 | NA | NA |
| cea2.i.48201 | Y80D3A.9 | 0.98 | 7.03 | 0.000622 | WBGene00013587 | Y80D3A.9 |
| cea2.p.119644 | F59D6.3 | 0.94 | 8.74 | 0.000645 | WBGene00019105 | F59D6.3 |
| cea2.3.30545 | Y73F8A.8 | 1.22 | 6.42 | 0.000718 | WBGene00004170 | pqn-90 |
| cea2.c.34112 | C24B9.3 | 0.97 | 9.59 | 0.000752 | WBGene00016048 | C24B9.3 |
| cea2.i.39301 | F47B8.5 | 0.86 | 7.63 | 0.000767 | WBGene00009806 | F47B8.5 |
| cea2.p.146457 | F52D1.3 | 0.91 | 7.86 | 0.000763 | WBGene00004127 | pqn-40 |
| cea2.d.08064 | T26E4.9 | 3.11 | 6.37 | 0.000774 | WBGene00012051 | T26E4.9 |
| cea2.c.26547 | C28C12.7b | 1.02 | 9.33 | 0.000832 | NA | NA |
| cea2.p.113075 | F15B9.1 | -1.2 | 9.65 | 0.000844 | WBGene00001387 | far-3 |
| cea2.p.61535 | F10F2.3 | 0.86 | 7.49 | 0.000878 | WBGene00008655 | lips-3 |
| cea2.d.13143 | C17C3.12b | -1.2 | 6.28 | 0.000927 | NA | NA |
| cea2.p.117903 | F53E10.4 | 1.35 | 7.43 | 0.00092 | WBGene00018760 | F53E10.4 |
| cea2.c.50260 | C49G7.4 | -3.3 | 5.62 | 0.000953 | WBGene00016782 | phat-3 |
