# Supplementary material for: Coordinate Regulation of Lipid Metabolism by Novel Nuclear Receptor Partnerships
Source: PLoS Genet. 2012 Apr 12;8(4):e1002645. doi: 10.1371/journal.pgen.1002645 (PMC3325191; doi:10.1371/journal.pgen.1002645)
Supplement: Table S6 — Occurrence of gene families in microarray results for NHR-66, based on GO terms. (DOC) [file pgen.1002645.s006.doc]

Table S6.

| GO Term | Description | P-value |
| --- | --- | --- |
| GO:0019673 | GDP-mannose metabolic process | 1.69E-04 |
| GO:0006005 | L-fucose biosynthetic process | 6.70E-04 |
| GO:0006004 | fucose metabolic process | 6.70E-04 |
| GO:0042354 | L-fucose metabolic process | 6.70E-04 |
| GO:0042353 | fucose biosynthetic process | 6.70E-04 |
| GO:0042350 | GDP-L-fucose biosynthetic process | 6.70E-04 |
| GO:0046368 | GDP-L-fucose metabolic process | 6.70E-04 |
| GO:0009226 | nucleotide-sugar biosynthetic process | 6.70E-04 |
| GO:0016540 | protein autoprocessing | 7.69E-04 |
| GO:0007367 | segment polarity determination | 7.69E-04 |
| GO:0007365 | periodic partitioning | 7.69E-04 |
