# Supplementary material for: Coordinate Regulation of Lipid Metabolism by Novel Nuclear Receptor Partnerships
Source: PLoS Genet. 2012 Apr 12;8(4):e1002645. doi: 10.1371/journal.pgen.1002645 (PMC3325191; doi:10.1371/journal.pgen.1002645)
Supplement: Table S7 — List of differentially expressed genes in nhr-80 compared to wild-type animals using microarray analysis. The data represent the analysis from four independent mRNA isolations and microarray hybridizations. “ID” refers to the identity of individual spots on the arrays, “logFC” represents the log of the fold change, “AveExpr” represents the averaged spot intensity. (DOC) [file pgen.1002645.s007.doc]

Table S7.

| Name | ID | logFC | AveExpr | P.Value | Gene WB ID | Gene Public Name |
| --- | --- | --- | --- | --- | --- | --- |
| F10D2.9 | cea2.c.35201 | -5.55 | 8.18 | 5.11E-08 | WBGene00001399 | fat-7 |
| W06D12.3 | cea2.c.39182 | -2.17 | 8.62 | 1.58E-06 | WBGene00001397 | fat-5 |
| T09F5.9 | cea2.p.124403 | -1.75 | 11.1 | 3.49E-06 | WBGene00011668 | clec-47 |
| F38E11.1 | cea2.i.25955 | -1.81 | 8.35 | 6.65E-06 | WBGene00002012 | hsp-12.3 |
| VZK822L.1 | cea2.c.31436 | -1.3 | 11 | 2.85E-05 | WBGene00001398 | fat-6 |
| F54B11.11 | cea2.i.54612 | -1.2 | 9.09 | 9.18E-05 | WBGene00010034 | F54B11.11 |
| F54E7.2 | cea2.c.19871 | 1.63 | 12.4 | 9.37E-05 | WBGene00004481 | rps-12 |
| K01G5.1 | cea2.c.21064 | 1.13 | 7.93 | 8.64E-05 | WBGene00010476 | tag-331 |
| K09H11.7 | cea2.d.33151 | -1.73 | 10.6 | 7.59E-05 | WBGene00019604 | K09H11.7 |
| ZK666.6 | cea2.i.18053 | -2.24 | 8.61 | 7.33E-05 | WBGene00014046 | clec-60 |
| F25H2.5 | cea2.c.02775 | 1.19 | 12.9 | 0.000145 | WBGene00009119 | F25H2.5 |
| Y39B6A.1 | cea2.p.127901 | -1.14 | 13 | 0.000134 | WBGene00012664 | Y39B6A.1 |
| F13B6.3 | cea2.p.90417 | -1.1 | 9.61 | 0.00016 | WBGene00017418 | F13B6.3 |
| F22A3.2 | cea2.p.142010 | -1.17 | 7.98 | 0.000202 | WBGene00017688 | ttr-35 |
| C54G6.5 | cea2.p.05632 | 1.2 | 10.5 | 0.000254 | WBGene00005002 | spp-17 |
| F15E11.14 | cea2.p.158386 | -1.04 | 10.2 | 0.000291 | WBGene00017500 | F15E11.14 |
| F17C11.6 | cea2.i.36871 | -1.08 | 9.37 | 0.000284 | WBGene00008917 | F17C11.6 |
| K07E8.3 | cea2.c.13321 | -1.06 | 11.3 | 0.000243 | WBGene00019495 | sdz-24 |
| Y18D10A.11 | cea2.p.19901 | 1.08 | 9.42 | 0.000262 | WBGene00012481 | Y18D10A.11 |
| K01G5.2a | cea2.d.04407 | 0.94 | 10.2 | 0.00037 | NA | NA |
| K01G5.5 | cea2.p.67570 | 0.94 | 10.1 | 0.000392 | WBGene00010478 | K01G5.5 |
| K02G10.7 | cea2.p.149038 | -1.19 | 8.74 | 0.000406 | WBGene00000176 | aqp-8 |
| M02D8.4b | cea2.d.33921 | -1.16 | 9.57 | 0.000385 | NA | NA |
| T21G5.3 | cea2.c.06057 | 1.47 | 11.1 | 0.000369 | WBGene00001598 | glh-1 |
| ZK593.3 | cea2.i.31671 | -0.98 | 11.3 | 0.000417 | WBGene00014003 | ZK593.3 |
| B0244.2 | cea2.c.16150 | -0.88 | 12.9 | 0.000546 | WBGene00002048 | ida-1 |
| C53A3.2 | cea2.i.35082 | -2.05 | 7.99 | 0.000482 | WBGene00016892 | C53A3.2 |
| F16F9.2 | cea2.p.141148 | -1.16 | 8.03 | 0.000616 | WBGene00001068 | dpy-6 |
| F21H7.4 | cea2.p.114258 | -0.93 | 8.23 | 0.000588 | WBGene00009030 | clec-233 |
| K01G5.10 | cea2.i.20094 | 1 | 8.06 | 0.000601 | WBGene00010481 | K01G5.10 |
| K01G5.7 | cea2.c.21122 | 1.61 | 12.6 | 0.00049 | WBGene00006536 | tbb-1 |
| Y34F4.2 | cea2.p.75989 | -1.3 | 5.8 | 0.000565 | WBGene00021337 | Y34F4.2 |
| Y45F10C.5 | cea2.d.44087 | -0.93 | 8.37 | 0.000522 | NA | NA |
| ZC395.5 | cea2.i.22724 | -2.59 | 8.64 | 0.000613 | WBGene00022597 | ZC395.5 |
| C32H11.12 | cea2.p.87235 | -1.09 | 7.33 | 0.000705 | WBGene00007875 | dod-24 |
| F56C9.11 | cea2.c.20147 | 0.88 | 8.47 | 0.000735 | WBGene00018954 | F56C9.11 |
| B0412.4 | cea2.c.16346 | 1.86 | 13.5 | 0.000816 | WBGene00004498 | rps-29 |
| C45G7.3 | cea2.d.16969 | -2.17 | 6.74 | 0.000841 | WBGene00016670 | ilys-3 |
| M02D8.4a | cea2.c.47012 | -1.09 | 10.1 | 0.000841 | NA | NA |
| M02D8.6 | cea2.c.47027 | -1.11 | 10.7 | 0.000843 | WBGene00019732 | M02D8.6 |
| F15E11.1 | cea2.d.20845 | -1.46 | 12.3 | 0.000936 | WBGene00017490 | F15E11.1 |
|  |  |  |  |  |  |  |
