# Supplementary material for: Coordinate Regulation of Lipid Metabolism by Novel Nuclear Receptor Partnerships
Source: PLoS Genet. 2012 Apr 12;8(4):e1002645. doi: 10.1371/journal.pgen.1002645 (PMC3325191; doi:10.1371/journal.pgen.1002645)
Supplement: Table S8 — Occurrence of gene families in microarray results for NHR-80, based on GO terms. (DOC) [file pgen.1002645.s008.doc]

Table S8.

| GO Term | Description | P-value |
| --- | --- | --- |
| GO:0006412 | translation | 2.40E-12 |
| GO:0034645 | cellular macromolecule biosynthetic process | 2.86E-11 |
| GO:0009059 | macromolecule biosynthetic process | 7.19E-11 |
| GO:0007275 | multicellular organismal development | 2.34E-06 |
| GO:0001676 | long-chain fatty acid metabolic process | 2.76E-06 |
| GO:0042759 | long-chain fatty acid biosynthetic process | 2.76E-06 |
| GO:0006633 | fatty acid biosynthetic process | 3.00E-06 |
| GO:0044249 | cellular biosynthetic process | 3.06E-06 |
| GO:0006869 | lipid transport | 1.19E-05 |
| GO:0006631 | fatty acid metabolic process | 1.41E-05 |
| GO:0009058 | biosynthetic process | 1.60E-05 |
| GO:0032787 | monocarboxylic acid metabolic process | 2.41E-05 |
| GO:0016053 | organic acid biosynthetic process | 7.00E-05 |
| GO:0046394 | carboxylic acid biosynthetic process | 7.00E-05 |
| GO:0007338 | single fertilization | 1.03E-04 |
| GO:0044283 | small molecule biosynthetic process | 1.43E-04 |
| GO:0051301 | cell division | 1.49E-04 |
| GO:0009631 | cold acclimation | 1.52E-04 |
| GO:0008610 | lipid biosynthetic process | 2.25E-04 |
| GO:0048609 | multicellular organismal reproductive process | 2.35E-04 |
| GO:0007276 | gamete generation | 2.50E-04 |
| GO:0012501 | programmed cell death | 2.67E-04 |
| GO:0006915 | apoptotic process | 2.72E-04 |
| GO:0008219 | cell death | 3.93E-04 |
| GO:0016265 | death | 3.93E-04 |
| GO:0010332 | response to gamma radiation | 4.71E-04 |
| GO:0009792 | embryo development ending in birth or egg hatching | 5.15E-04 |
| GO:0009790 | embryo development | 6.10E-04 |
| GO:0022414 | reproductive process | 7.02E-04 |
| GO:0006030 | chitin metabolic process | 7.21E-04 |
| GO:0043436 | oxoacid metabolic process | 8.05E-04 |
| GO:0006082 | organic acid metabolic process | 8.05E-04 |
| GO:0019752 | carboxylic acid metabolic process | 8.05E-04 |
| GO:0000910 | cytokinesis | 9.35E-04 |
| GO:0042180 | cellular ketone metabolic process | 9.38E-04 |
