# Supplementary material for: Coordinate Regulation of Lipid Metabolism by Novel Nuclear Receptor Partnerships
Source: PLoS Genet. 2012 Apr 12;8(4):e1002645. doi: 10.1371/journal.pgen.1002645 (PMC3325191; doi:10.1371/journal.pgen.1002645)
Supplement: Table S9 — List of common genes regulated by NHR-49 and NHR-66 and list of common genes regulated by NHR-49 and NHR-80 (a) Genes common to NHR-49 and NHR-66 (b) Genes common to NHR-49 and NHR-80. (DOC) [file pgen.1002645.s009.doc]

Table S9a.

| Gene | Gene Name |
| --- | --- |
| ZK218.5 | Secreted surface protein |
| B0222.4 | Sphingosine phosphate lyase |
| T05B4.3 | Pharyngeal toxin related-4 |
| Y65B4BR.1 | Phospholipase |
| B0348.2 | Unnamed protein |
| ZK617.2 | Lipase-related |
| F27E5.1 | Acid ceramidase |
| B0454.8 |  |
| T19D12.4 |  |
| W02B12.1 | Phospholipase |
| C29F3.5 | c-type lectin |
| C47A10.1 | P-glycoprotein related |
| E02H9.5 | Glycosyl hydrolase |
| F53E10.4 | Infection response gene |

Table S9b.

| Gene | Gene Name |
| --- | --- |
| K09H11.7 |  |
| C53A3.2 |  |
| C32H11.12 | Dod-24, downstream of DAF-16 |
