# Supplementary material for: Coordinate Regulation of Lipid Metabolism by Novel Nuclear Receptor Partnerships
Source: PLoS Genet. 2012 Apr 12;8(4):e1002645. doi: 10.1371/journal.pgen.1002645 (PMC3325191; doi:10.1371/journal.pgen.1002645)
Supplement: Table S10 — Summary of lifespan data. (DOC) [file pgen.1002645.s010.doc]

Table S10.

| Strain | Mean+/-SEM (days) | 50^th^ percentile (days) | No. of animals | p-value (compared to WT) |
| --- | --- | --- | --- | --- |
| WT | 17.35+/-0.34 | 18 | 196 | N/A |
| *nhr-49(nr2041)* | 9.52+/-0.23 | 9.5 | 139 | <0.0001 |
| *nhr-66(ok940)* | 17.16+/-0.41 | 17 | 96 | ns (0.73) |
| *nhr-80(tm1011)* | 13.19+/-0.38 | 12.5 | 129 | <0.0001 |
| *nhr-13 (gk796)* | 14.17+/-0.4 | 13 | 135 | <0.0001 |
| *nhr-80; nhr-13* | 12.29+/-0.37 | 11 | 139 | <0.0001 |
