# Supplementary material for: Coordinate Regulation of Lipid Metabolism by Novel Nuclear Receptor Partnerships
Source: PLoS Genet. 2012 Apr 12;8(4):e1002645. doi: 10.1371/journal.pgen.1002645 (PMC3325191; doi:10.1371/journal.pgen.1002645)
Supplement: Table S11 — Lifespan and relative C18:0 fatty acid abundance. (DOC) [file pgen.1002645.s011.doc]

Table S11.

| Strain | Mean Lifespan | % of total fatty acids  C18:0 C18:1n9 | | Ratio |
| --- | --- | --- | --- | --- |
| WT | 17.35+/-0.34 | 4.98+/-0.15 | 5.14+/-0.22 | 0.98+/-0.06 |
| *nhr-49(nr2041)* | 9.52+/-0.23 | 8.97+/-0.62 | 2.46+/-0.39 | 3.74+/-0.33 |
| *nhr-66(ok940)* | 17.16+/-0.41 | 5.68+/-0.35 | 5.23+/-0.49 | 1.09+/-0.04 |
| *nhr-80(tm1011)* | 13.19+/-0.38 | 8.63+/-0.48 | 3.6+/-0.17 | 2.4+/-0.22 |
| *nhr-13(gk796)* | 14.17+/-0.4 | 5.27+/-0.24 | 5.46+/-0.13 | 0.96+/0.02 |
| *nhr-80; nhr-13* | 12.29+/-0.37 | 9.74+/-0.33 | 3.32+/-0.27 | 2.99+/-0.22 |
